# Laser-Assisted Ultrafast Fabrication of Crystalline Ta-Doped TiO_2_ for High-Humidity-Processed Perovskite Solar Cells

**DOI:** 10.1021/acsami.1c24225

**Published:** 2022-03-25

**Authors:** Hongbo Mo, Dong Wang, Qian Chen, Wei Guo, Suresh Maniyarasu, Andrew G. Thomas, Richard J. Curry, Lin Li, Zhu Liu

**Affiliations:** † Department of Materials, The University of Manchester, Oxford Road, Manchester M13 9PL, U.K.; ‡ Laser Processing Research Center, Department of Mechanical, Aerospace and Civil Engineering, The University of Manchester, Oxford Road, Manchester M13 9PL, U.K.; § Photon Science Institute, Department of Electrical and Electronic Engineering, The University of Manchester, Oxford Road, Manchester, M13 9PL, U.K.; ∥ Department of Physics and Astronomy, School of Natural Sciences, The University of Manchester, Oxford Road, Manchester M13 9PL, U.K.; ⊥ Henry Royce Institute, The University of Manchester, Oxford Road, Manchester M13 9PL, U.K.

**Keywords:** laser-assisted doping, Ta-doped TiO_2_, ambient-processed, perovskite solar cells

## Abstract

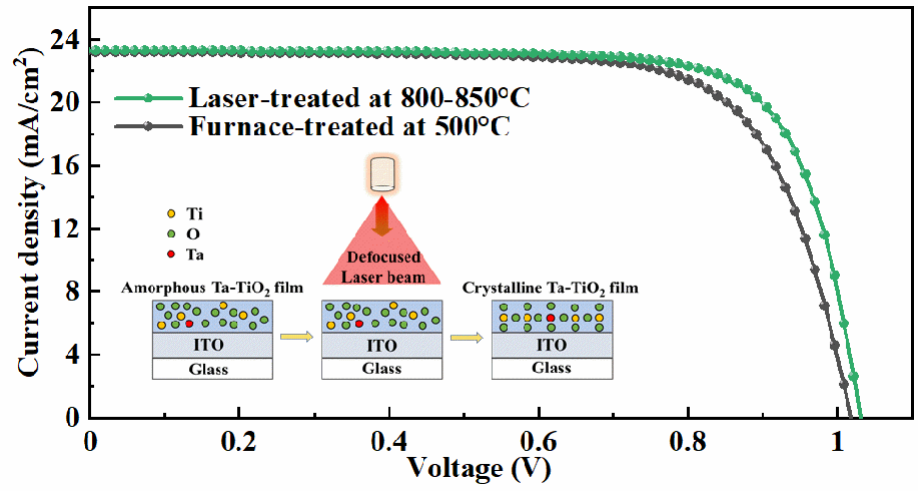

A titanium dioxide (TiO_2_) compact film is a widely used electron transport layer (ETL) for n–i–p planar perovskite solar cells (PSCs). However, TiO_2_ sufferers from poor electrical conductivity, leading to high energy loss at the perovskite/ETL/transparent conductive oxide interface. Doping the TiO_2_ film with alkali- and transition-metal elements is an effective way to improve its electrical conductivity. The conventional method to prepare these metal-doped TiO_2_ films commonly requires time-consuming furnace treatments at 450–600 °C for 30 min to 3 h. Herein, a rapid one-step laser treatment is developed to enable doping of tantalum (Ta) in TiO_2_ (Ta-TiO_2_) and to simultaneously induce the crystallization of TiO_2_ films from its amorphous precursor to an anatase phase. The PSCs based on the Ta-TiO_2_ films treated with the optimized fiber laser (1070 nm) processing parameters (21 s with a peak processing temperature of 800–850 °C) show enhanced photovoltaic performance in comparison to that of the device fabricated using furnace-treated films at 500 °C for 30 min. The ambient-processed planar PSCs fabricated under high relative humidity (RH) of 50–70% display power conversion efficiencies (PCEs) of 18.34% and 16.04% for devices based on Cs_0.1_FA_0.9_PbI_3_ and CH_3_NH_3_PbI_3_ absorbers, respectively. These results are due to the improved physical and chemical properties of the Ta-TiO_2_ films treated by the optimal laser process in comparison to those for the furnace process. The laser process is rapid, simple, and potentially scalable to produce metal-doped TiO_2_ films for efficient PSCs.

## 1 Introduction

Perovskite solar cells (PSCs) constitute a promising technology in the renewable energy field due to their excellent photovoltaic performance, low cost, ease of fabrication, and scalability.^1^ The certified power conversion efficiency (PCE) of PSCs has increased rapidly from 3.9% to over 25.5% within the past decade.^2,3^ In these devices the electron transport layer (ETL) plays a vital role in effectively extracting photogenerated electrons and blocking holes that enable an efficient electron collection at the ETL/perovskite interface for high-performance PSCs.^4^

Titanium dioxide (TiO_2_) has been widely used as the ETL for PSCs and dye-sensitized solar cells due to its excellent chemical stability, low cost, high optical transparency, and reasonable charge transport ability.^5−9^ TiO_2_ also shows a suitable band alignment with the perovskite layer due to its conduction band minimum lying at lower energy than that of the perovskite absorber.^10^ For planar PSCs with an n–i–p architecture, a compact TiO_2_ film is deposited between the perovskite layer and a transparent conductive oxide (TCO) to transport the photogenerated electrons and suppress charge recombination. To date, planar PSCs based on the compact TiO_2_ films have achieved PCEs of over 21%.^11,12^

Despite these advantages and the promising results of using TiO_2_ as an ETL, TiO_2_ suffers from a low electrical conductivity that impedes its use for state of the art planar PSCs. Surface modification is an effective method for passivating TiO_2_ surface trap states. Various materials, including fullerene (C_60_),^13^ fullerene derivatives,^14,15^ small-molecule materials such as an HOCO-R-NH_3_^+^I^–^ anchor group,^16^ and dopamine^17^ have been used to adjust the photocarrier dynamics at the interface. On the other hand, doping TiO_2_ with transition- and alkali-metal elements is a direct and efficient way to enhance its electrical conductivity. So far, various metal elements, including lithium (Li), tantalum (Ta), zinc (Zn), neodymium (Nd), niobium (Nb), cobalt (Co), aluminum (Al), magnesium (Mg), europium (Eu), yttrium (Y), and cesium (Cs), have been doped within a compact or mesoporous TiO_2_ film to improve the electrical conductivity and electron transport in the TiO_2_ ETL.^10,18−26^

Conventional methods to prepare these doped TiO_2_ films commonly require a time-consuming furnace-annealing process at 450–600 °C for 30 min to 3 h,^18−21,27^ with issues such as bending of the glass substrates due to the long thermal process of over 500 °C being reported.^28^ To date, only a few studies have investigated alternative methods to assist the doping of metal elements in TiO_2_ for PSCs. Recently, the chemical deposition method at low temperature were applied for doping of Sn, Zn, and Ta/Nb in TiO_2_, which provided a potential method for preparing TiO_2_-based flexible PSCs.^29−32^ In addition, a rapid flame-annealing doping method with a 40 s processing time was developed to assist in the doping of Co in TiO_2_ for enhanced performance of PSCs. The researchers suggested that a high processing temperature of up to 1000 °C with the flame-annealing process potentially improved the doping quality of the TiO_2_ films.^33,34^ To the best of our understanding, no work has been reported to use a laser-assisted doping process to fabricate doped-TiO_2_ films for PSCs.

Here we report a rapid one-step laser process to assist in the doping of Ta in TiO_2_ and simultaneously crystallize the TiO_2_ films from its amorphous precursor to the anatase phase. In comparison with the conventional furnace treatment of 500 °C for 30 min, we prepared Ta-doped TiO_2_ (Ta-TiO_2_) through a laser-assisted method with laser processing times ranging from 16 to 25 s and peak processing temperatures from 600 to 950 °C. The physical and chemical properties of Ta-TiO_2_ films prepared by furnace and laser treatments were systematically studied. The photovoltaic performances of planar PSCs fabricated with the furnace- and laser-treated Ta-TiO_2_ films were investigated, and compared with those of CH_3_NH_3_PbI_3_ and Cs_0.1_FA_0.9_ PbI_3_ perovskite absorbers. Device fabrication processes were undertaken in ambient air with a high relative humidity (RH) of 50–70%. The green antisolvent ethyl acetate (EA) was used in a one-step deposition process to assist in the fabrication of high-quality perovskite films under ambient conditions.

## 2 Results and Discussion

**Figure 1:**
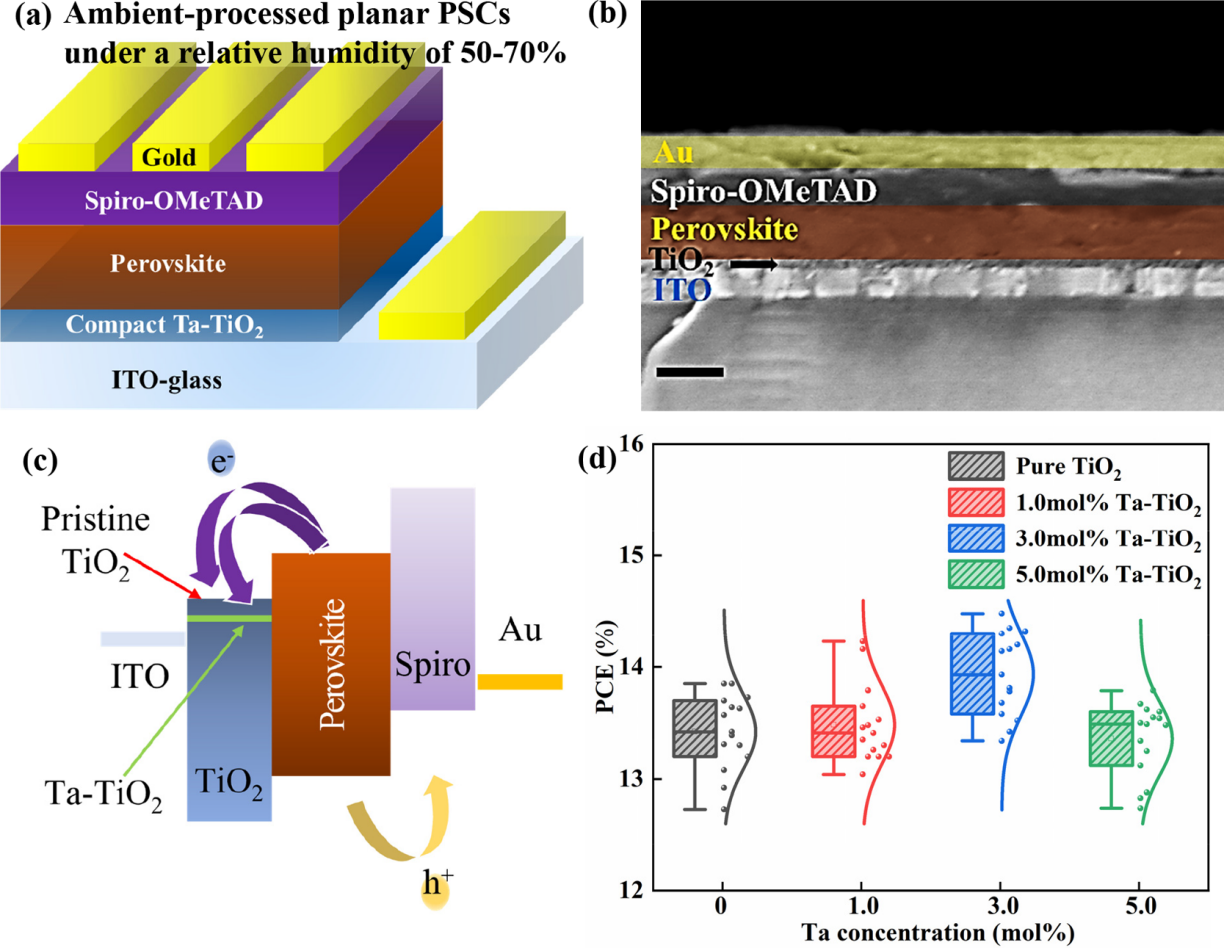
(a) Schematics representation of the air-processed planar PSCs under high RH of 50–70%. (b) Cross-sectional view of the air-processed planar PSC (scale bar  200 nm). (c) Band diagram of the planar PSCs based on the TiO_2_ and Ta-TiO_2_ ETLs. (d) PCE distribution of planar PSCs based on pristine TiO_2_ and Ta-TiO_2_ films with various doping ratios.

To identify the optimal doping ratio of Ta in a compact TiO_2_ film, we first investigated the photovoltaic performance of planar PSCs based on these films as a function of Ta concentration. Figure 1a gives a schematic representation of the planar PSCs assembled in this study with an ITO-glass/TiO_2_/perovskite/Spiro-OMeTAD/Au architecture. A cross-sectional view of the actual PSCs is shown in Figure 1b. The thicknesses of compact TiO_2_ film, perovskite layer, Spiro-OMeTAD, and Au electrodes are approximately 40, 150, 90, and 90 nm, respectively. The perovskite used here was CH_3_NH_3_PbI_3_ with 0.1 M PbCl_2_ additive with detail of the fabrication process provided in the Experimental Section. Apart from the thermal evaporation of Au electrodes, all fabrication processes were performed in ambient air with a relative humidity of 50–70%, as shown in Figure S1 of the Supporting Information.

Various molar ratios of tantalum butoxide (1.0, 3.0, and 5.0 mol %) were added to TiO_2_ precursors to induce the Ta doping in TiO_2_ films. Previous studies found that doping of Ta in TiO_2_ could improve the conductivity and cause the conduction band and Fermi level of the TiO_2_ to shift downward, which can improve the electron injection from the perovskite to TiO_2_ and improve the PCEs of the PSCs, as presented in Figure 1c.^18,35,36^ To verify the findings and find the optimized doping ratio, we compared the PCEs of planar PSCs based on pristine TiO_2_ and Ta-TiO_2_ with various doping ratios (1.0, 3.0, and 5.0 mol %), as shown in Figure 1d. A summary of the photovoltaic parameters is presented in Table S1. We find that the average *J*_SC_ values of the PSCs increased from 19.1 to 20.5 mA cm^–2^ with the increased doping of Ta from 0 to 5 mol %, as shown in Figure S2a. However, the average *V*_OC_ and fill factor (FF) of the PSCs decreased from 1.019 to 0.975 V and from 69.01% to 66.97%, respectively, with the increased doping of Ta, as shown in Figure S2b,c. The optimized Ta doping ratio in this study is found to be 3 mol % with an average PCE of 14.01% in comparison to the pristine PSCs with an average PCE of 13.42%. The typical current density–voltage (*J–V*) curves of pristine and PSCs with various Ta doping ratios are presented in Figure S2d.

To confirm the Ta doping of the TiO_2_ films and understand the cause of the improvement in device performance, we performed Raman spectroscopy, X-ray diffraction (XRD), X-ray photoelectron spectroscopy (XPS), photoluminescence (PL) spectroscopy, and resistance measurements. Figure S3a,b gives the Raman spectra of pristine TiO_2_ and Ta-TiO_2_ films with various doping ratios. All pristine TiO_2_ and Ta-TiO_2_ films show the anatase phase of TiO_2_ with peaks assigned at 144 cm^–1^ (E_g_*), 399 cm^–1^ (B_1g_*), and 639 cm^–1^ (E_g_*).^37^ The *E*_g_ peak at 144 cm^–1^ shows a shift toward a higher wavenumber and a decreased peak intensity with an increase in the Ta doping ratio. This finding potentially indicates that the Ta dopant is incorporated into the TiO_2_, which agrees with previous studies.^18,38^

The XRD patterns in Figures S3c and S4a further confirm the findings from Raman spectroscopy. All pristine TiO_2_ and Ta-TiO_2_ films show peaks at 25.3° corresponding to TiO_2_ anatase planes (101), and no peaks corresponding to the rutile phase are observed. We noticed that the peaks corresponding to anatase planes (101) at 25.3° shift toward lower 2θ values with an increase in the Ta doping ratios. This is possibly due to a larger ionic radius of Ta^5+^ (0.64 Å) in comparison to Ti^4+^ (0.61 Å), causing an expansion of the TiO_2_ lattice.^18^ The shifted peaks from XRD patterns indicate that the Ta element might be doped into the TiO_2_ crystal lattice. In addition, we noticed that the peak intensity at anatase planes (101) decreases with an increase in the Ta doping ratio. The full width at half-maximum (fwhm) for the peaks at anatase planes (101) increases from 0.7822° to 0.9552° with an increase in the Ta doping ratio, indicating a decrease in the crystallinity and grain size of the TiO_2_ films, as shown in Table S2.

To further confirm the doping of Ta into the TiO_2_ crystal lattice, an XPS analysis was performed to study the change in surface chemistry after doping. Figure S3d shows the high-resolution XPS spectra of Ta peaks for pristine TiO_2_ and Ta-TiO_2_ films, while Figure S4b shows the XPS survey spectra. We observed two peaks located at 26.4 and 28.2 eV, corresponding to Ta 4f_7/2_ and 4f_5/2_. These binding energies are consistent with the presence of Ta^5+^.^35^ The Ta peaks of XPS spectra further confirmed that Ta is successfully incorporated into TiO_2_ as a dopant. The peak intensity for Ta 4f increases with an increase in the Ta doping ratio from 3 to 5 mol %. Figure S4c,d shows the high-resolution XPS spectra for the pristine TiO_2_ and 3 mol % Ta-TiO_2_ films with Ti 2p_3/2_ and 2p_1/2_ peaks at 458.8 and 464.5 eV, corresponding to Ti^4+^. We notice that a small shoulder appeared at 457.1 eV corresponding to Ti^3+^ for the 3 mol % Ta-TiO_2_. This is due to the formation of Ta^5+^ that potentially introduces more Ti^3+^ in the lattice.^18^

To investigate the effect of Ta doping on the optical properties of the Ta-TiO_2_ films, we used ultraviolet–visible–near-infrared (UV–vis–NIR) spectroscopy, as shown in Figure S4e. All pristine and Ta-TiO_2_ films show a high transmission over the visible region. We noticed that the film transmission at around 370–400 nm increases slightly with an increase in Ta doping ratio. The band gaps for the Ta-TiO_2_ films calculated using Tauc plots decrease with an increase in Ta doping ratio, as shown in Figure S4f. These results are in good agreement with previous studies on doping Ta in TiO_2_ films.^18,36^

To investigate the effect of Ta doping on the electrical conductivity of the TiO_2_ film, we measured the current–voltage (*I–V*) curves for the devices based on an ITO/TiO_2_/Au configuration, as shown in Figure S3e. The slope of the *I–V* curve is in proportion to the electrical conductivity of the TiO_2_ film.^36^ We observed an increase in the TiO_2_ conductivity with an increase in the Ta doping ratio, and the highest conductivity achieved was with the 5 mol % Ta-TiO_2_. PL spectroscopy was used to evaluate the effect of Ta doping on the electron transfer from the perovskite film to the TiO_2_ films using an ITO/TiO_2_/perovskite configuration, as shown in Figure S3f. We noticed that the sample based on the 3 mol % Ta-TiO_2_ shows the strongest quenching effect of the PL spectra. These findings are consistent with the previous photovoltaic measurements which showed that the device based on 3 mol % Ta-TiO_2_ shows the highest PCE. Although 5 mol % Ta-TiO_2_ shows the highest conductivity, the high doping ratio reduces the crystallinity as well as potentially introduces more Ti^3+^ in the TiO_2_ lattice that could act as recombination sites to reduce the charge transport ability.^39^

**Figure 2:**
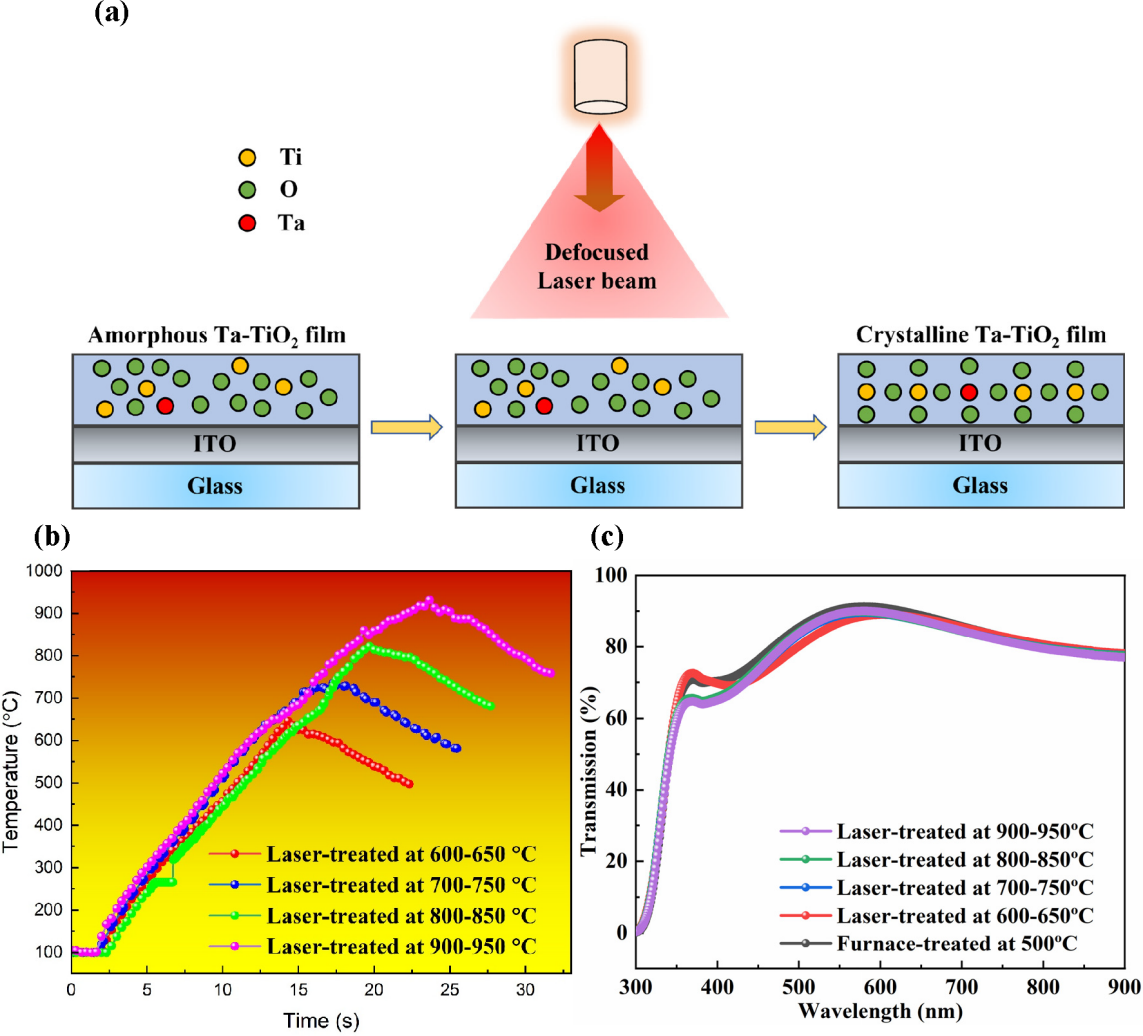
(a) Schematic representation of the one-step laser process to assist the doping of Ta in TiO_2_ and crystallizing the TiO_2_ film from its amorphous precursor to the anatase phase simultaneously. (b) Temperature profiles of the 3 mol % Ta-TiO_2_ films during laser treatment with various laser parameters. (c) UV–vis–NIR transmission spectra of the 3 mol % Ta-TiO_2_ films treated as a function of temperature.

Having determined an optimized doping ratio of 3 mol % Ta in the TiO_2_ films, we then carried out a rapid one-step laser process to assist the doping of Ta and induce the crystallization of the TiO_2_ films simultaneously from its amorphous precursor to the anatase phase. A schematic representation of the laser process is shown in Figure 2a. To investigate the temperature changes of the Ta-TiO_2_ films during the laser processing, we used a high-resolution FLIR IR thermal camera to record the thermal profiles of the Ta-TiO_2_ films under various laser processing parameters. As shown in Figure 2b, we noticed that the peak processing temperatures of 600–650, 700–750, 800–850, and 900–950 °C were achieved with continuous-wave (CW) 1070 nm laser irradiation for 16, 17, 21, and 25 s, respectively. The thermal profiles of the Ta-TiO_2_ coated on the ITO glass with peak processing temperatures of 600–650, 700–750, 800–850, and 900–950 °C recorded by a thermal camera during the laser treatments are shown in Figure S5. The detailed laser processing parameters are presented in Table S3.

To compare the optical properties of the Ta-TiO_2_ films fabricated with the furnace and laser processes with various laser processing parameters, we used UV–vis–NIR spectroscopy, as shown in Figure 2c. All furnace- and laser-treated Ta-TiO_2_ films show a high transmission over the visible region, ideal for use as the ETLs for n–i–p PSCs. In addition, we noticed that the film transmission at around 370 nm decreases slightly with an increase of peak processing temperature. The band gaps of Ta-TiO_2_ films calculated using Tauc plots are similar for all furnace- and laser-treated Ta-TiO_2_ films with various laser parameters, as shown in Figure S6.

**Figure 3:**
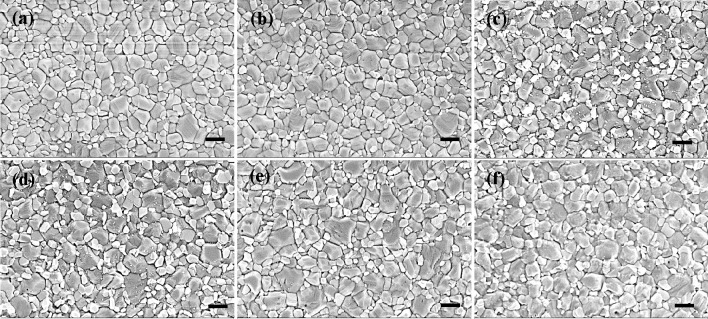
Top view SEM images of the perovskite films deposited on (a) a furnace-treated pristine TiO_2_ film, (b) a furnace-treated 3 mol % Ta-TiO_2_ film, and laser-treated Ta-TiO_2_ films with peak processing temperatures of (c) 600–650 °C, (d) 700–750 °C, (e) 800–850 °C, and (f) 900–950 °C (scale bar   500 nm).

To probe the grain size and surface coverage of the perovskite films deposited on the pristine TiO_2_ and Ta-TiO_2_ films treated with different treatments, we used scanning electron microscopy (SEM) to investigate the morphology of perovskite films deposited on the TiO_2_ and Ta-TiO_2_ films. All pristine TiO_2_ and Ta-TiO_2_ films present a good surface coverage of the perovskite films and similar grain sizes of around 0.15–0.16 μm^2^, as shown in Figure 3 and Figure S7. The surface morphologies of the pristine TiO_2_ and Ta-TiO_2_ films were also investigated, as shown in Figure S8. All of the furnace- and laser-treated pristine TiO_2_ and Ta-TiO_2_ films show the typical morphology of crystalline compact TiO_2_ films. However, we found that the laser-treated Ta-TiO_2_ film with a peak processing temperature of 900–950 °C (Figure S8f) shows more pinholes in comparison to the other films. These pinholes might act as charge recombination sites that impede the charge transfer of the photogenerated electrons.

**Figure 4:**
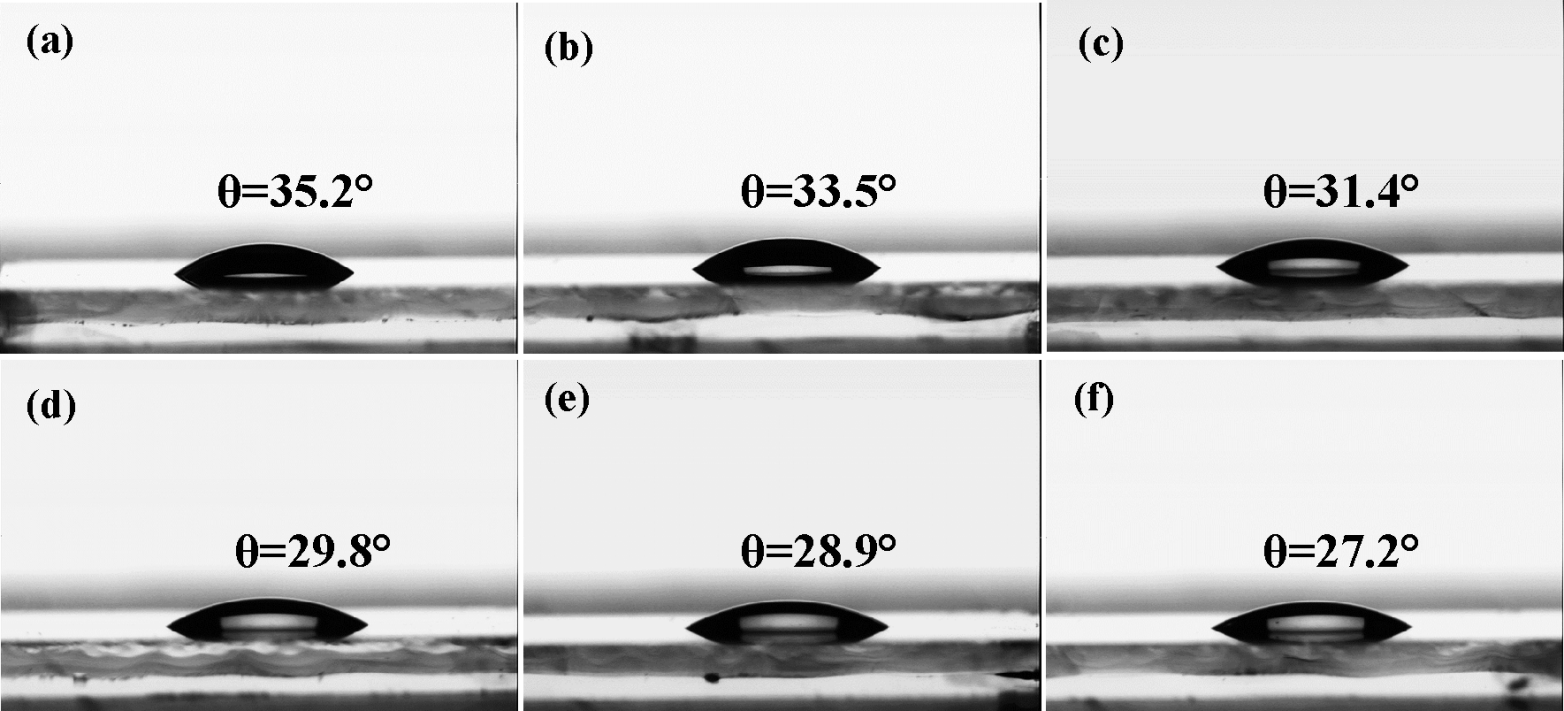
Water contact measurements of (a) a furnace-treated pristine TiO_2_ film, (b) a furnace-treated 3 mol % Ta-TiO_2_ film, and laser-treated 3 mol % Ta-TiO_2_ films with the peak processing temperatures of (c) 600–650 °C, (d) 700–750 °C, (e) 800–850 °C, and (f) 900–950 °C.

To investigate the surface wettability of pristine TiO_2_ and Ta-TiO_2_ films, we measured the contact angle of these films. We notice a smaller contact angle for the 3 mol % Ta-TiO_2_ in comparison to the pristine TiO_2_, as shown in Figure 4. With the laser treatments, the contact angle further decreases with an increase in the laser peak processing temperatures. The laser-treated Ta-TiO_2_ film with a peak temperature of 900–950 °C shows the smallest contact angle of 27.2° in comparison to the furnace-treated pristine TiO_2_ and Ta-TiO_2_ films with contact angles of 35.2° and 33.5°, respectively. A smaller contact angle indicates a better wettability that enables the perovskite precursor to spread well on the TiO_2_ film and promote the heterogeneous nucleation and more uniform growth of the perovskite crystals due to a lower nucleation barrier for the growth of perovskite crystals on the more hydrophilic surface.^40,41^ Therefore, a better surface wettability of the Ta-TiO_2_ films treated by the laser process could improve the contact at the TiO_2_/perovskite interface, promote a more uniform growth of the perovskite crystals, and reduce the formation of pinholes and defects.^42−44^

**Figure 5:**
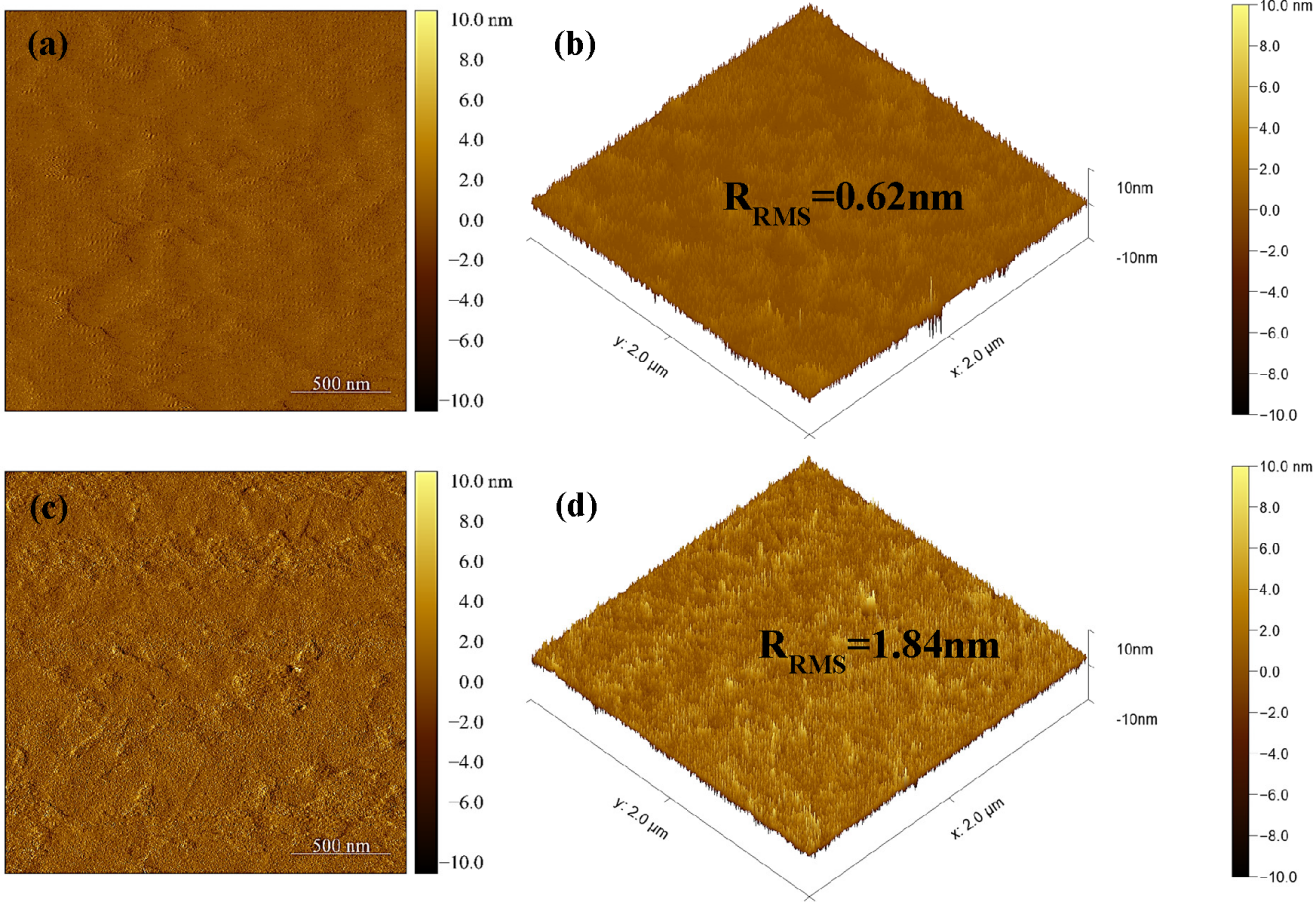
(a, b) AFM topography for the furnace-treated 3 mol % at 500 °C. (c, d) AFM topography for the laser-treated Ta-TiO_2_ film with a peak processing temperature of 800–850 °C (scale bar 500 nm).

To understand the cause for the improvement in the surface wettability of the Ta-TiO_2_ film treated by the laser process, we used atomic force microscopy (AFM) to compare the surface roughnesses of the Ta-TiO_2_ films treated with the furnace and laser processes. The Ta-TiO_2_ film treated with a laser for 21 s with a peak temperature of 800–850 °C shows a root-mean-square (RMS) surface roughness of 1.84 nm, higher than that of the Ta-TiO_2_ film treated with a furnace at 500 °C for 30 min with an RMS value of 0.62 nm, as shown in Figure 5. For the same material with a contact angle lower than 90°, its wettability increases with an increase in surface roughness due to the enlargement of the contact area of the surface.^45^ This is in good agreement with our finding that the rougher surface of the laser-treated Ta-TiO_2_ film provides a better wettability in comparison to the furnace-treated Ta-TiO_2_ film with lower surface roughness.

**Figure 6:**
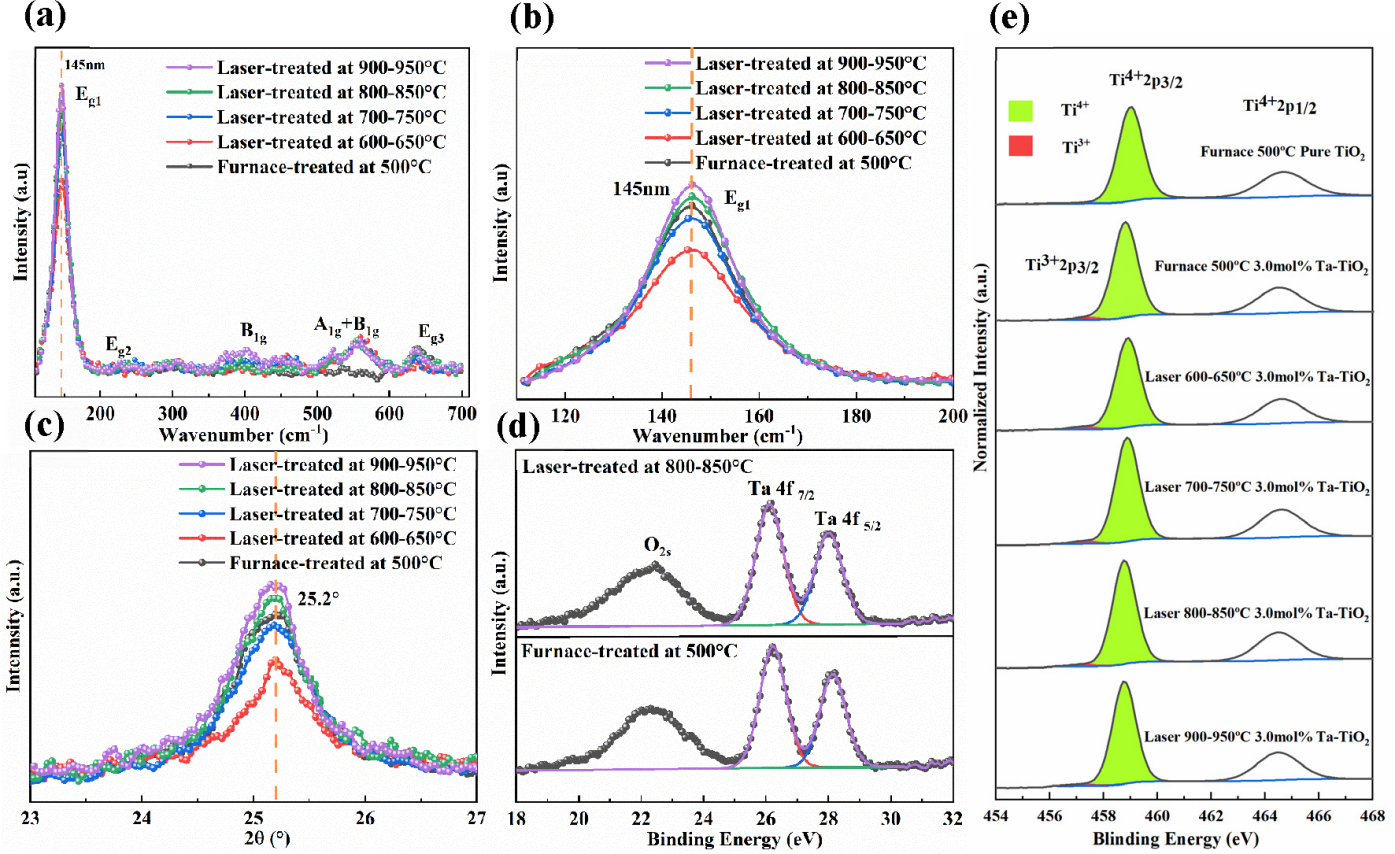
(a, b) Raman spectra of the furnace- and laser-treated Ta-TiO_2_ films as a function of temperature. (c) XRD patterns of the furnace- and laser-treated Ta-TiO_2_ films as a function of temperature. (d) High-resolution XPS spectra in the Ta 4f region for Ta-TiO_2_ films treated with a furnace at 500 °C and laser films treated with a peak processing temperature of 800–850 °C. (e) Ti 2p core-level XPS spectra of Ta-TiO_2_ films treated under various conditions, with the 2p_3/2_ peaks fitted into Ti^4+^ (green) and Ti^3+^ (red) components located at 458.8 and 457.3 eV, respectively. All of the peaks are normalized to identical intensities for comparison.

To further study the difference between the furnace- and laser-treated Ta-TiO_2_ films, we conducted a comprehensive analysis to probe the physical and chemical changes of the Ta-TiO_2_ films. Figure 6a,b shows the Raman spectra of the furnace- and laser-treated Ta-TiO_2_ films. We noticed that all samples present the anatase phase of the TiO_2_ films. The laser-treated Ta-TiO_2_ films show an increase in Raman peak intensity with an increase in the laser processing temperature. The Ta-TiO_2_ films treated with the laser processes with peak processing temperatures of 800–850 and 900–950 °C show higher Raman peak intensities at the E_g_ peak (145 cm^–1^). These results indicate that higher laser processing temperatures enhance the crystallinity of the Ta-TiO_2_ film. It is also worth noting that the E_g1_ peak remains at a similar position for the Ta-TiO_2_ film processed by both furnace and laser-assisted methods with different processing temperatures. These results indicate that the processing methods and temperatures in this study do not necessarily affect the Ta doping concentration in the TiO_2_ lattice.

The XRD patterns further confirm that all furnace- and laser-treated Ta-TiO_2_ films are in the anatase form, as shown in Figure 6c and Figure S9. We noticed that the intensity for the peak corresponding to anatase planes (101) at 25.2° increases with an increase in laser processing temperature. The films treated with the laser processes with the peak processing temperatures of 800–850 and 900–950 °C also show higher peak intensity at 25.2° in comparison to the furnace-treated Ta-TiO_2_ film. In addition, the peak corresponding to anatase planes (101) at 25.2° remains at similar positions for the Ta-TiO_2_ film processed by both furnace- and laser-assisted methods with different processing temperatures. These results agree with the previous Raman results (Figure 6a,b) that the use of a laser process could enhance the crystallinity of the Ta-TiO_2_ film but not necessarily affect the Ta doping concentration in the TiO_2_ lattice. The fwhm of the peaks at 25.2° and crystallite sizes calculated from the Scherrer equation are summarized in Table S4. In addition, although TiO_2_ starts to convert from the anatase to the rutile phase at around 600 °C with the furnace treatment, no peaks corresponding to the rutile phase were found for laser treatment from both Raman spectra and XRD patterns, even with a peak processing temperature of 900–950 °C.^46^ This is possibly due to the fact that the rapid laser treatments for all processes were completed within 25 s.

To study the change in the surface chemistry for the furnace- and laser-treated Ta-TiO_2_ films, we performed an XPS analysis, as shown in Figure 6d,e. Two peaks at 26.4 and 28.2 eV corresponding to Ta 4f_7/2_ and 4f_5/2_ confirm that the Ta was successfully incorporated into the TiO_2_ crystal lattice. We calculated the Ta^5+^/Ti^4+^ ratios for the furnace- and laser-treated Ta-TiO_2_ films, as shown in Table S5. Both furnace- and laser-treated Ta-TiO_2_ films show similar Ta^5+^/Ti^4+^ ratios. These results are in good agreement with the previous results from Raman spectroscopy (Figure 6a,b) and XRD measurements (Figure 6c and Figure S9) that the use of the laser process in this study does not necessarily affect the Ta doping concentration in the TiO_2_ lattice.

We then calculated the Ti^3+^/Ti^4+^ ratios for the furnace- and laser-treated Ta-TiO_2_ films, as shown in Table S5. We observe a decrease in the Ti^3+^/Ti^4+^ ratio with an increase in laser processing temperature. As was previously reported, the presence of oxygen in ambient air during the annealing process could promote the oxidation of Ti^3+^ to Ti^4+^ in the TiO_2_ film.^47,48^ The laser-treated Ta-TiO_2_ samples with peak processing temperaturea of 800–850 and 900–950 °C show a lower surface Ti^3+^ concentration in comparaison to the furnace-treated sample. This is possibly due to a more efficient oxidation of the Ta-TiO_2_ in ambient air with the high-temperature laser treatment as well as a more effective removal of the organic residues from the Ta-TiO_2_ precursor.^49,50^ Previous studies suggested that the Ti^3+^ defects acting as the defects at the surface of TiO_2_ hinder the electron transport and increase the chances of charge recombination, thus leading to a reduced photovoltaic performance of the PSCs.^51,52^ Therefore, a lower concentration of Ti^3+^ defects on the surface of Ta-TiO_2_ treated with peak laser processing temperatures of 800–850 and 900–950 °C might contribute to a better device performance in comparison to the furnace-treated Ta-TiO_2_.

To probe the distribution of the Ta dopants in the TiO_2_ films, we used transmission electron microscopy (TEM) with energy-dispersive X-ray (EDX) analysis. Figure S10a shows the high-resolution cross-sectional view of the Ta-TiO_2_ films treated with a peak laser processing temperature of 800–850 °C. The thickness of the Ta-TiO_2_ films is around 40–50 nm. We note that the Ta dopants are uniformly distributed across the TiO_2_ films from the EDX elemental mapping results, as shown in Figure S10b–d.

**Figure 7:**
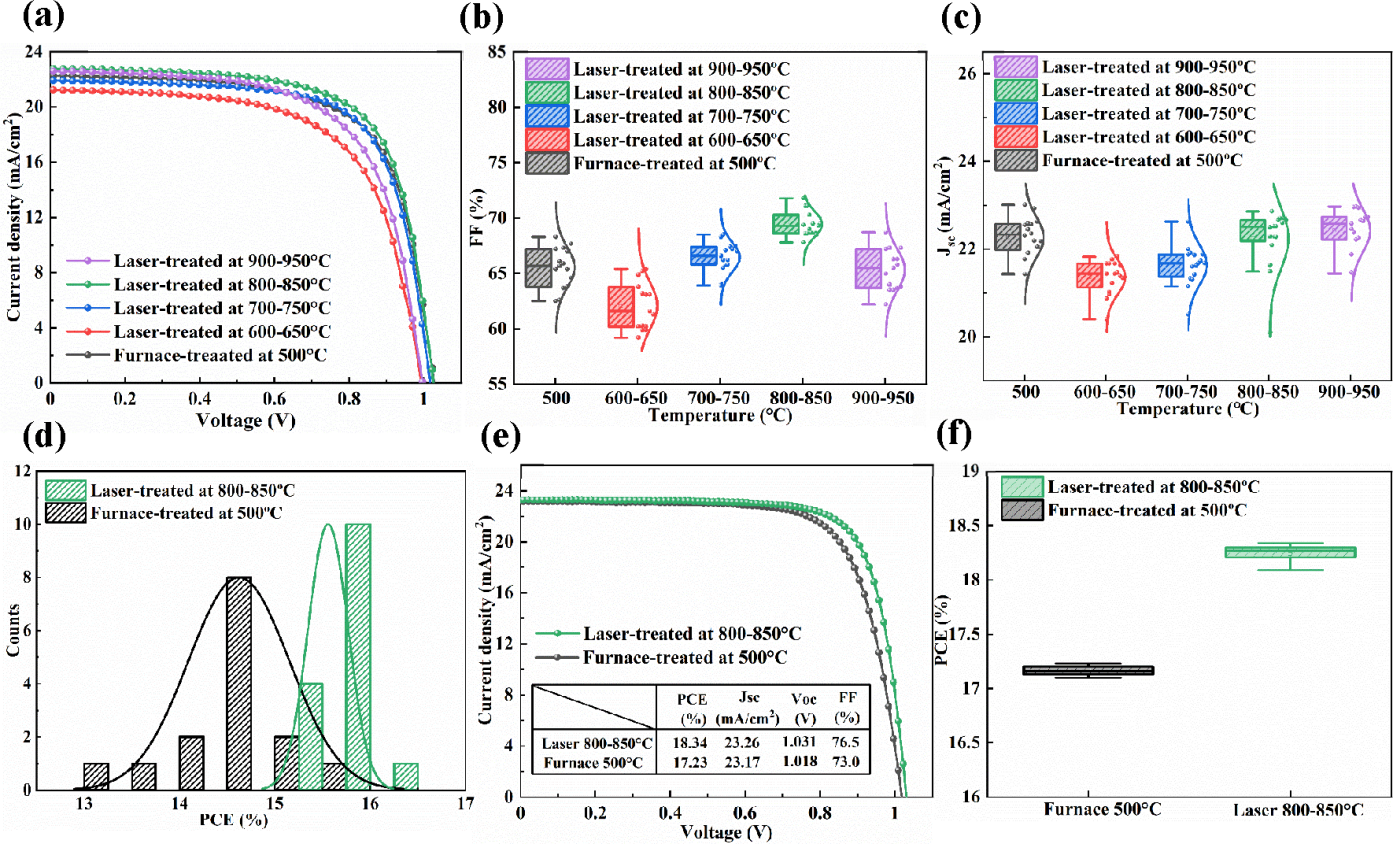
(a) Typical *J–V* curves for air-processed CH_3_NH_3_PbI_3_ devices based on the furnace- and laser-treated Ta-TiO_2_ films as a function of temperature. (b) FF and (c) *J*_SC_ distribution for CH_3_NH_3_PbI_3_ devices based on the furnace- and laser-treated Ta-TiO_2_ films as a function of temperature. (d) PCE and count distribution for CH_3_NH_3_PbI_3_ devices based on the Ta-TiO_2_ films treated with furnace and optimized laser processing parameters. (e) Typical *J–V* curves for air-processed Cs_0.1_FA_0.9_PbI_3_ devices based on the Ta-TiO_2_ films treated with furnace and optimized laser processes. (f) PCE distribution for Cs_0.1_FA_0.9_PbI_3_ devices based on the Ta-TiO_2_ films treated with furnace and optimized laser processes.

To examine whether the laser-treated Ta-TiO_2_ films improve the device performance, we assembled planar PSCs with two types of perovskite absorbers based on an ITO-glass/TiO_2_/perovskite/Spiro-OMeTAD/Au architecture. Figure 7a shows the typical *J–V* curves for the devices based on the CH_3_NH_3_PbI_3_ with 0.1 M Pb(SCN)_2_ additive. The detailed fabrication process is described in the Experimental Section. A summary of the photovoltaic parameters for the samples treated under different conditions is given in Table S6. We observe that the device PCE increases at first with increasing peak laser processing temperature from 600–650 to 800–850 °C but then drops at a peak temperature of 900–950 °C, as shown in Figure S11. The increase of the peak laser processing temperature from 600–650 to 800–850 °C mainly contributes to a higher FF and *J*_SC_, as shown in Figure 7b,c. This improvement is possibly due to a reduction in the surface Ti^3+^ defect concentration and better crystallinity and wettability for the Ta-TiO_2_ films treated at higher peak laser processing temperature, as supported by previous characterizations and measurements. The laser-treated devices with the peak processing temperature of 800–850 °C show a higher average PCE of 15.56% and a champion PCE of 16.04% in comparison the furnace-treated devices with an average PCE of 14.62% and a champion PCE of 15.52%, as shown in Figure 7d and Table S6. In addition, there is a noticeable drop in the FF for the devices with a laser peak processing temperature at 900–950 °C in comparison to that for the 800–850 °C treatment, which leads to lower PCEs. Therefore, the optimized laser parameter is with the peak processing temperature at 800–850 °C.

To further confirm the improvement in the photovoltaic performance, we assembled planar PSCs based on a Cs_0.1_FA_0.9_ PbI_3_ absorber to compare the differences between the devices based on the furnace-treated Ta-TiO_2_ films and those based on the laser-treated films with the optimized parameter. Figure 7e shows the typical *J–V* curves for the devices based on the Ta-TiO_2_ films treated with a furnace at 500 °C for 30 min and a laser with a peak processing temperature of 800–850 °C for 21 s. The laser-treated devices with the optimized parameter show a higher average PCE of 18.24% and a champion PCE of 18.34% in comparison to the furnace-treated devices with an average PCE of 17.16% and a champion PCE of 17.23%, mainly due to the noticeably higher FF for the laser-treated devices in comparison to that of the furnace-treated devices. A summary of the photovoltaic parameters is presented in Figure 7f and Table S7. These results agree with previous studies which show that a better crystallinity and wettability, in conjunction with the reduced concentration of surface Ti^3+^ defects for the Ta-TiO_2_ films treated with the optimized laser parameter, contribute to an enhanced photovoltaic performance.^39,51,52^ A summary of the photovoltaic performances of several recent ambient-processed PSCs in comparison to that in the current work is given in Table S8.

**Figure 8:**
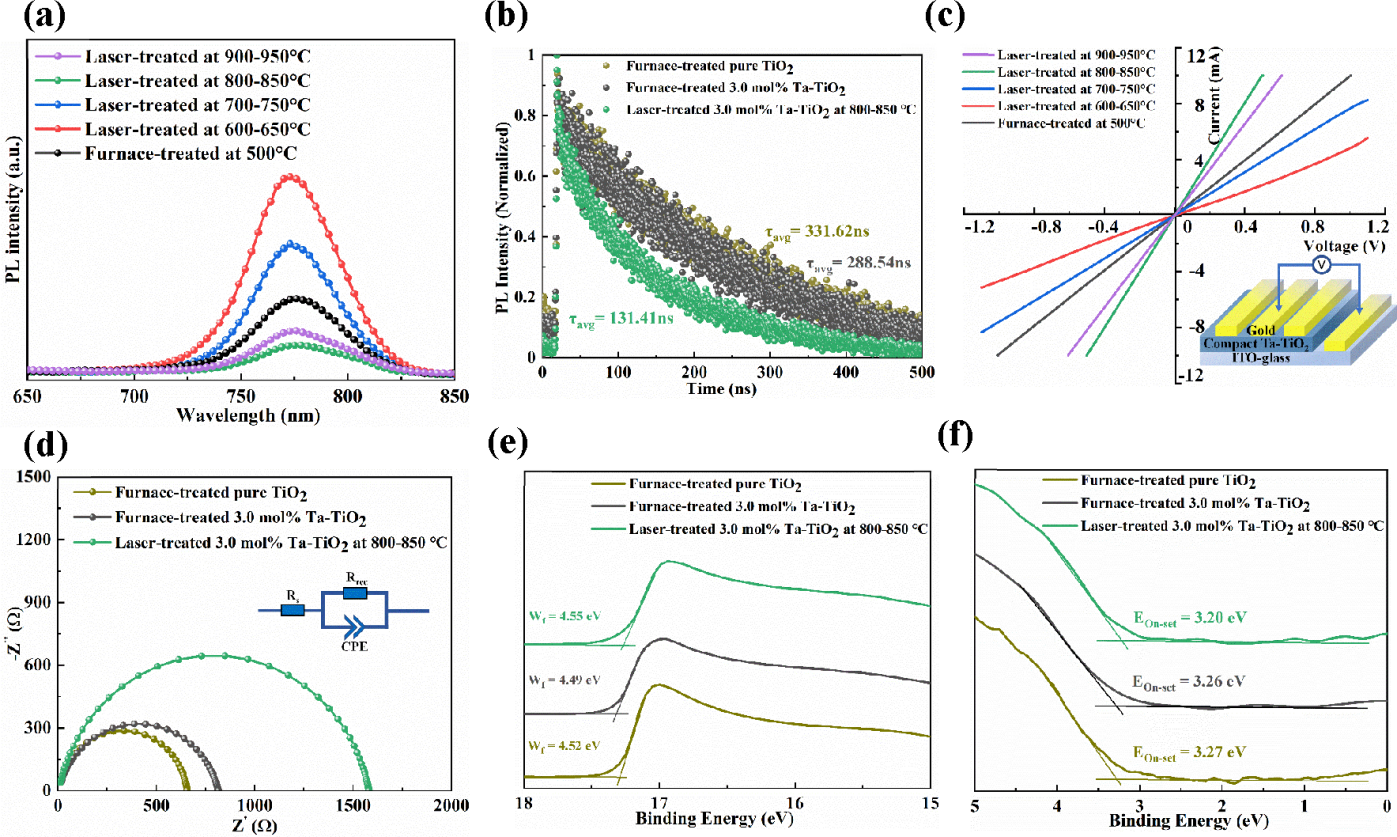
(a) Steady-state PL spectra for the perovskite films coated on the furnace- and laser-treated Ta-TiO_2_ films as a function of temperature. (b) Time-resolved PL curves of perovskite films deposited on furnace-treated pure TiO_2_ and 3.0 mol % Ta-TiO_2_ at 500 °C and laser-treated 3.0 mol % Ta-TiO_2_ at 800–850 °C. (c) *I–V* curves for the devices based on the furnace- and laser-treated Ta-TiO_2_ films with an ITO/TiO_2_/Au configuration as a function of temperature. (d) Nyquist plots of devices based on furnace-treated pure TiO_2_ and 3.0 mol % Ta-TiO_2_ at 500 °C and laser-treated 3.0 mol % Ta-TiO_2_ at 800–850 °C. The inset shows the equivalent circuit of the Nyquist plot. (e, f) UPS [He II] spectra of furnace-treated pure TiO_2_ and 3.0 mol % Ta-TiO_2_ at 500 °C and laser-treated 3.0 mol % Ta-TiO_2_ at 800–850 °C with cutoff energies (*E*_cut-off_) and on-set energies (*E*_on-set_), respectively.

To further evaluate the differences between the Ta-TiO_2_ films treated under different conditions, we performed a series of analyses and measurements to understand the cause for the improvement in the device performance by the laser treatment. Figure 8a shows the PL spectra for samples based on the furnace- and laser-treated Ta-TiO_2_ films with an ITO/Ta-TiO_2_/perovskite configuration. We noticed that the quenching effect of the PL spectra increases with an increase in peak laser processing temperature from 600–650 to 800–850 °C and eventually drops at 900–950 °C. These results are consistent with the photovoltaic measurements that the optimized laser processing condition has a peak processing temperature of 800–850 °C. The laser-treated Ta-TiO_2_ film with a peak processing temperature of 800–850 °C also shows a stronger quenching effect than the furnace-treated Ta-TiO_2_ film, indicating a potential improvement in the charge transport ability of the ETL. One of the causes for the laser-treated Ta-TiO_2_ film with a peak temperature of 900–950 °C showing a lower PL quenching effect than that of the 800–850 °C could be due to the presence of more pinholes on the Ta-TiO_2_ film treated at 900–950 °C on the basis of the SEM observation (Figure S8f) that impedes the electron transfer and thus PL quenching.

Figure 8b shows the normalized time-resolved photoluminescence (TRPL) spectra of a perovskite on various ETLs, and Table S9 summarizes the lifetime and the corresponding amplitudes. The lifetime decay curves consist of a fast decay component τ_1_ and a slow decay component τ_2_. In general, τ_1_ results from the quenching of charge carriers at the interface, whereas τ_2_ results from the radiative recombination of free charge carriers due to traps in the bulk.^43^ The sample based on furnace-treated pure TiO_2_ had the longest average lifetime τ_avg_ of 331.62 ns, whereas τ_avg_ values of samples based on furnace-treated and laser-treated 3.0 mol % Ta-TiO_2_ decrease to 288.54 and 131.41 ns, respectively. In addition, the fast-decay component τ_1_ decreased from 10.19 ns with furnace-treated TiO_2_ to 5.02 and 5.01 ns with furnace-treated and laser-treated 3.0 mol % Ta-TiO_2_, respectively. The decreased τ_avg_ and τ_1_ values indicate that electrons can be extracted efficiently from the perovskite layer to the TiO_2_ film with low recombination loss in the bulk and at the interface.

To understand the film electrical conductivity differences with different treatments, we measured the *I–V* curves for the devices based on the furnace- and laser-treated Ta-TiO_2_ films with an ITO/TiO_2_/Au configuration, as shown in Figure 8c. We noticed that the slope of the *I–V* curve increases with an increase in the peak laser processing temperature from 600–650 to 800–850 °C and then decreases at 900–950 °C. These results indicate that the electrical conductivity of the Ta-TiO_2_ film increases with the peak laser processing temperature up to 800–850 °C and then decreases at 900–950 °C. In addition, the laser-treated Ta-TiO_2_ film with a peak processing temperature of 800–850 °C has a higher electrical conductivity in comparison to the furnace-treated Ta-TiO_2_ film. These findings agree with the previous PL, photovoltaic, and material characterizations and measurements. The Ta-TiO_2_ film treated under the optimized laser condition with improved film crystallinity, wettability, and reduced surface Ti^3+^ defects contributes to an improved electrical conductivity and charge transport ability of the ETLs.

To investigate the effect of Ta doping and laser treatment in TiO_2_ on charge transport, we used electrochemical impedance spectroscopy (EIS). The corresponding Nyquist plots and EIS fitting parameters of devices based on furnace-treated pure TiO_2_ and 3.0 mol % Ta–TiO_2_ and laser-treated 3.0 mol % Ta–TiO_2_ with the equivalent circuit are shown in Figure 8d and Table S10. The devices based on laser-treated 3.0 mol % Ta-TiO_2_ exhibit a lower series resistance (*R*_s_) of 3.0 Ω and a significantly larger recombination resistance (*R*_rec_) of 1578.0 Ω in comparison to the devices based on furnace-treated pure TiO_2_ with an *R*_s_ value of 5.3 Ω and *R*_rec_ value of 651.1 Ω and 3.0 mol % Ta-TiO_2_ with an *R*_s_ value of 3.3 Ω and *R*_rec_ value of 811.7 Ω, respectively. The EIS indicates that the laser-assisted doping method contributes to suppressing charge recombination.

It is worth mentioning that, although the laser-treated Ta-TiO_2_ film with a peak processing temperature of 900–950 °C shows slightly better crystallinity in comparison to that of the 800–850 °C film, it has a lower electrical conductivity and weaker quenching effect of the PL spectra in comparison to those of the 800–850 °C film. To further understand the cause of these results, we measured the resistance of the ITO electrodes after different treatments. A two-probe method was used to measure the ITO resistance with a fixed distance of 17 mm between the two points of the electrode. A schematic representation of the resistance measurement with an Ossila eight-pixel substrate is shown in Figure S12. We found that the ITO resistance increases with an increase in the peak laser processing temperature, as shown in Figure S13 and Table S11. This is due to the transformation of ITO crystallinity to equiaxial nanograins that increases the resistance of the ITO with a high-temperature treatment.^53^ This could be another reason that the devices with a peak laser processing temperature of 900–950 °C has a lower photovoltaic performance in comparison to that of the 800–850 °C film.

In addition, although the ITO resistances for the samples treated with laser processing temperatures of 600–650 and 700–750 °C are lower than that of the 800–850 °C sample, they have lower photovoltaic performances, due to the noticeably lower film crystallinity and greater number of surface Ti^3+^ defects in comparison to those of the 800–850 °C sample. In comparison to furnace-treated Ta-TiO_2_ films at 500 °C for 30 min, all laser-treated samples show lower ITO resistance, due to the more rapid laser processing time in comparison to that for the furnace process. Therefore, it could be a combination of improved crystallinity, wettability, and reduced surface Ti^3+^ defects of the Ta-TiO_2_ film and lower ITO resistance treated with the optimized laser parameter that contributes to a better device performance in comparison to the furnace-treated samples.

To determine the influence of laser-assisted tantalum doping on the energy level positions of the TiO_2_ films, we used ultraviolet photoelectron spectroscopy (UPS). Figure 8e,f illustrates the magnified UPS spectra of the valence band edge (*E*_on-set_) and secondary electron cutoff edge (*E*_cut-off_), respectively. The Fermi level (*E*_F_) and valence band maximum (*E*_VBM_) can be determined by *E*_F_ = E_cut-off_ – 40.8 eV (photo energy of He II) + 18.99 eV (bias voltage energy) and *E*_VBM_ = *E*_F_ – *E*_on-set_. The conduction band minimum (*E*_CBM_) can be determined from *E*_VBM_ and band gap (*E*_g_) by *E*_CBM_ = *E*_VBM_ + *E*_g_.^54^ The energy band diagram and the corresponding results are displayed in Figure S14 (the specific calculation results are given in Table S12 in the Supporting Information). The *E*_VBM_ values of both laser-treated and furnace-treated 3.0 mol % Ta-TiO_2_ films are calculated to be 7.75 eV, while the *E*_VBM_ value of furnace-treated pure TiO_2_ films is 7.79 eV. The *E*_CBM_ values of laser-treated and furnace-treated 3.0 mol % Ta-TiO_2_ films are calculated to be 4.21 and 4.20 eV, respectively, while the *E*_CBM_ value of furnace-treated pure TiO_2_ films is 4.22 eV. The results indicate that doping TiO_2_ with tantalum lowers the electron transport layer’s conduction band, promoting electron extraction from the perovskite to TiO_2_ films, and the laser-treated Ta-TiO_2_ film has an energy level position similar to that of the furnace-treated Ta-TiO_2_.

## 3 Conclusion

In summary, we have demonstrated a rapid one-step laser process to assist in the doping of Ta in TiO_2_ and simultaneous crystallization of the TiO_2_ films from its amorphous precursor to the anatase phase. We have assessed the effect of the peak laser annealing temperature on the Ta-TiO_2_ crystallinity, wettability, surface chemistry, electrical conductivity, photovoltaic performance, and ITO resistance in comparison to the furnace-treated samples. As a result, the Ta-TiO_2_ films treated with the optimized laser parameter with a peak processing temperature of 800–850 °C for 21 s show improved film quality and photovoltaic performance for the air-processed planar PSCs based on both CH_3_NH_3_PbI_3_ and Cs_0.1_FA_0.9_ PbI_3_ perovskite absorber in comparison to the furnace-treated films at 500 °C for 30 min. These results are supported by evidence from XRD, Raman, SEM, TEM, AFM, UV–vis-NIR XPS, UPS, PL, TRPL, and EIS and resistance and photovoltaic measurements. This laser process potentially opens a new avenue for the rapid fabrication of high-quality doped-TiO_2_ for PSCs.

## 4 Experimental Section

### 4.1 Materials

Prepatterned eight-pixel ITO glass, methylammonium iodide (MAI; 98%), formamidinium iodide (FAI; 98%), 2,2′,7,7′-tetrakis[*N*,*N*-bis(4-methoxyphenyl)amino]-9,9′-spirobifluorene (spiro-OMeTAD; 99.0%) were purchased from Ossila. 1-Butanol (99.8%), titanium diisopropoxide bis(acetylacetonate) (75 wt % in isopropanol), tantalum(V) butoxide (99.99%), lead iodide (PbI_2_; 99.9985%), lead(II) chloride (PbCl_2_; 99.999%), lead(II) thiocyanate (Pb(SCN)_2_; 99.5%), ethyl acetate (99.8%), cesium iodide (CsI; 99.999%), dimethyl sulfoxide (DMSO; 99.9%), dimethylformamide (DMF; 99.8%), chlorobenzene (99.9%), 4-*tert*-butylpyridine (tBP; 98%), bis(trifluoromethane)sulfonimide lithium salt (LiTFSI; 99.95%), tris(2-(1*H*-pyrazol-1-yl)-4-*tert*-butylpyridine)cobalt(III) tris[bis(trifluoromethane)sulfonimide] (KF209; 98%), and acetonitrile (99.8%) were purchased from Sigma-Aldrich. Hellmanex III detergent was purchased from Alfa Aesar.

### 4.2 Device Fabrication

Prepatterned indium tin oxide (ITO) glasses were washed in an ultrasonic bath, in the sequence 3% Hellmanex solution in deionized water, ethanol, and finally deionized water, respectively. The substrates were then treated with UV–ozone for 15 min. The compact TiO_2_ layers were fabricated in the same way as in our previous method.^49^ The substrate was then annealed at 500 °C for 30 min in a furnace and finally cooled for 3 h to complete the furnace process. For Ta-doped solar cells, a certain amount of tantalum(V) butoxide was added to the TiO_2_ precursor with different molar ratios of Ta/Ti (1.0, 3.0, and 5.0 mol %). The same furnace process was applied to prepare Ta-doped TiO_2_ compact layers as for the pristine TiO_2_ films.

### 4.3 Laser Annealing

The setup of the laser process was based on our previous studies.^37,49^ An IPG fiber laser with a wavelength of 1070 nm was used in this study to carry out the laser-assisted doping process, with a defocused spot size of 19.6 cm^2^ (5 cm in diameter). A FLIR high-resolution IR thermal camera was used to record the temperature profiles during the laser annealing processes. For the ramping power program, during the high-power irradiation process, the laser output power was set to be 110, 113, 113, and 110 W cm^–2^ for irradiation times of 13, 14, 18, and 22 s to reach the peak temperatures of 600–650, 700–750, 800–850, and 900–950 °C, respectively. We noticed a small temperature leap at around 7 s for the thermal measurement of laser-treated Ta-TiO_2_ with a peak temperature of 800–850 °C. This is due to the occasional lag of the software but does not affect the overall temperature measurement. For the soaking irradiation process, the laser output powers were 26, 33, 36, and 36 W cm^–2^ for 3 s to stabilize the peak temperatures of the substrates at around 600–650, 700–750, 800–850, and 900–950 °C for 3 s, respectively. To adjust the IR emissivity of the different materials, we performed the adjustment using a thermocouple and a hot plate to measure the temperature of the materials and adjust the emissivity in the thermal recording software.

The CH_3_NH_3_PbI_3_ perovskite films were prepared by an antisolvent method on the basis of previous studies.^55−57^ A 461 mg portion of PbI_2_ and 28 mg of PbCl_2_ or 32 mg of Pb(SCN)_2_ were dissolved in 175 μL of DMSO and 825 μL of DMF, and the mixture was then stirred at 70 °C for 1 h. After the mixture was cooled to room temperature, 190 mg of MAI was added to the lead iodide solution to form the perovskite precursor. Before the deposition of the perovskite precursor, the TiO_2_ or Ta-TiO_2_ coated ITO glass substrates were preheated at 70 °C for 10 min. An 80 μL portion of the prepared perovskite precursor was then spin-coated on the preheated substrates at 4000 rpm for 30 s, and 200 μL of ethyl acetate was dripped on the substrate from 6 to 8 s after starting the spin coating program. The substrate was then annealed at 120 °C for 10 min to crystallize the perovskite film.

The Cs_0.1_FA_0.9_ PbI_3_ perovskite film was prepared by a similar antisolvent method. A 460 mg portion of PbI_2_ was dissolved in 200 μL of DMSO and 800 μL of DMF, and the mixture was then stirred at 70 °C for 1 h. After the the mixture was cooled to room temperature, 155 mg of FAI and 26 mg of CsI were added to the solution and the mixture was stirred at room temperature overnight. A 90 μL portion of the prepared perovskite precursor was then spin-coated on the preheated substrates at 1000 rpm for 10 s and 4000 rpm for 30 s. A 200 μL portion of ethyl acetate was dripped on the substrate 10 s before the end of the spin-coating program. The substrate was then annealed at 100 °C for 10 min to crystallize the perovskite film.

A 80 μL portion of Spiro-MeOTAD solution, consisting of 43 mg of spiro-MeOTAD, 10 μL of Li-TSFI (520 mg of Li-TSFI in 1 mL of acetonitrile), 14.5 μL of KF209 (300 mg KF209 in 1 mL of acetonitrile) and 15 μL of 4-*tert*-butylpyridine in 0.5 mL of chlorobenzene, was spin-coated on the perovskite film at 4000 rpm for 30 s. Finally, 80 nm of the gold layer was deposited via thermal evaporation. Apart from thermal evaporation, all processes were carried out in ambient air with an RH of 50–70%.

### 4.4 Material and Device Characterization

The morphologies of TiO_2_ films and the coated perovskite films were observed through a field-emission scanning electron microscope equipped with an in-lens detector (Ultra-55, Carl Zeiss). The Raman spectra of the TiO_2_ films were characterized by a Renishaw Raman spectrometer with a 514 nm excitation Ar^+^ laser. The XRD patterns of the TiO_2_ films were measured using a PANalytical XRD2 diffractometer. The UV–vis–NIR spectra of TiO_2_ films were acquired using a Shimadzu UV-2401PC spectrophotometer. The *J–V* curves for the devices were measured using an Oriel solar simulator at 100 mW cm^–2^ (AM 1.5G) connected to a Keighley 2420 source meter. The solar simulator was calibrated using an NREL-certified reference cell. A square metal mask with an area of 0.024 cm^2^ was used to determine the effective area under illumination. The water contact angle of TiO_2_ films was measured using an FTA188 analyzer. The surface roughness of the films was measured with a Bruker Multimodal 8 atomic force microscope (AFM) in tapping mode. The TEM and EDX for the films were captured by a Cs-corrected FEI Titan G2 80-200 S/TEM ChemiSTEM operating at 200 kV equipped with a high-efficiency Super-X EDS detector. The PL spectra and TRPL spectra of the films were measured using an FLS980 spectrometer (Edinburgh Instruments) with an excitation wavelength of 450 nm. The resistance of the ITO electrodes was measured with a two-probe method with a fixed distance of 17 mm between the two electrodes. The surface chemistry of the films was examined using a Kratos Axis Ultra X-ray Photoelectron Spectrometer equipped with a monochromated Al Kα X-ray source with a photon energy of 1486.7 eV. All XPS data were analyzed using CasaXPS software.^58^ UPS data were recorded with a Kratos Axis Ultra X-ray Photoelectron Spectrometer. The UPS experiments were carried out by employing a helium discharge lamp with an emission energy of 40.8 eV for He (II) emission, and −18.99 V was applied for the SEED edge. EIS was performed in the dark using a 0.9 V bias and a 10 mV signal with a frequency range of 100 Hz to 100 kHz.

## Supporting Information Available

Digital images, photovoltaic parameters, XPS, UV–vis–NIR, TEM, and SEM images, schematic diagrams, resistance measurements, thermal camera recordings, Raman and XRD results, efficiency comparison table, and other associated tables (PDF)
