# Supplementary material for: Laser-Assisted Ultrafast Fabrication of Crystalline Ta-Doped TiO2 for High-Humidity-Processed Perovskite Solar Cells
Source: ACS Appl Mater Interfaces. 2022 Mar 25;14(13):15141–53. doi: 10.1021/acsami.1c24225 (PMC9098116; doi:10.1021/acsami.1c24225)
Supplement: Supplementary file 1 — am1c24225_si_001.pdf [file am1c24225_si_001.pdf]

Supporting Information

Laser-assisted ultrafast fabrication of crystalline Ta-doped TiO_2 for high-humidity processed perovskite solar cells

Hongbo Mo,[ab] Dong Wang,[a] Qian Chen,[ab]* Wei Guo,[b] Suresh Maniyarasu,[cd] Andrew G Thomas,[ace] Richard J Curry,[ce] Lin Li[b] and Zhu Liu [ab]*

[a] Department of Materials, The University of Manchester, Oxford Road, Manchester, M13 9PL, UK

[b] Laser Processing Research Center, Department of Mechanical, Aerospace and Civil Engineering, The University of Manchester, Oxford Road, Manchester, M13 9PL, UK

[c] Photon Science Institute, Department of Electrical and Electronic Engineering, The University of Manchester, Oxford Road, Manchester, M13 9PL, UK

[d] Department of Physics and Astronomy, School of Natural Sciences, The University of Manchester, Oxford Road, Manchester M13 9PL, UK

[e] Henry Royce Institute, The University of Manchester, Oxford Road, Manchester M13 9PL, UK

* Corresponding authors: qian.chen-2@manchester.ac.uk, zhu.liu@manchester.ac.uk

No. of pages: 16

No. of figures: 14

No. of tables: 12

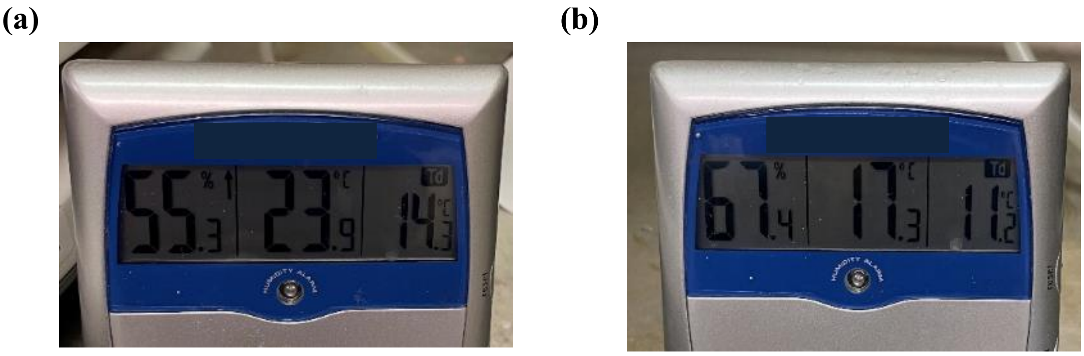

Figure S1 Humidity meter showing relative humidity of (a) 55.3% and (b) 67.4% during the fabrication of the PSCs.

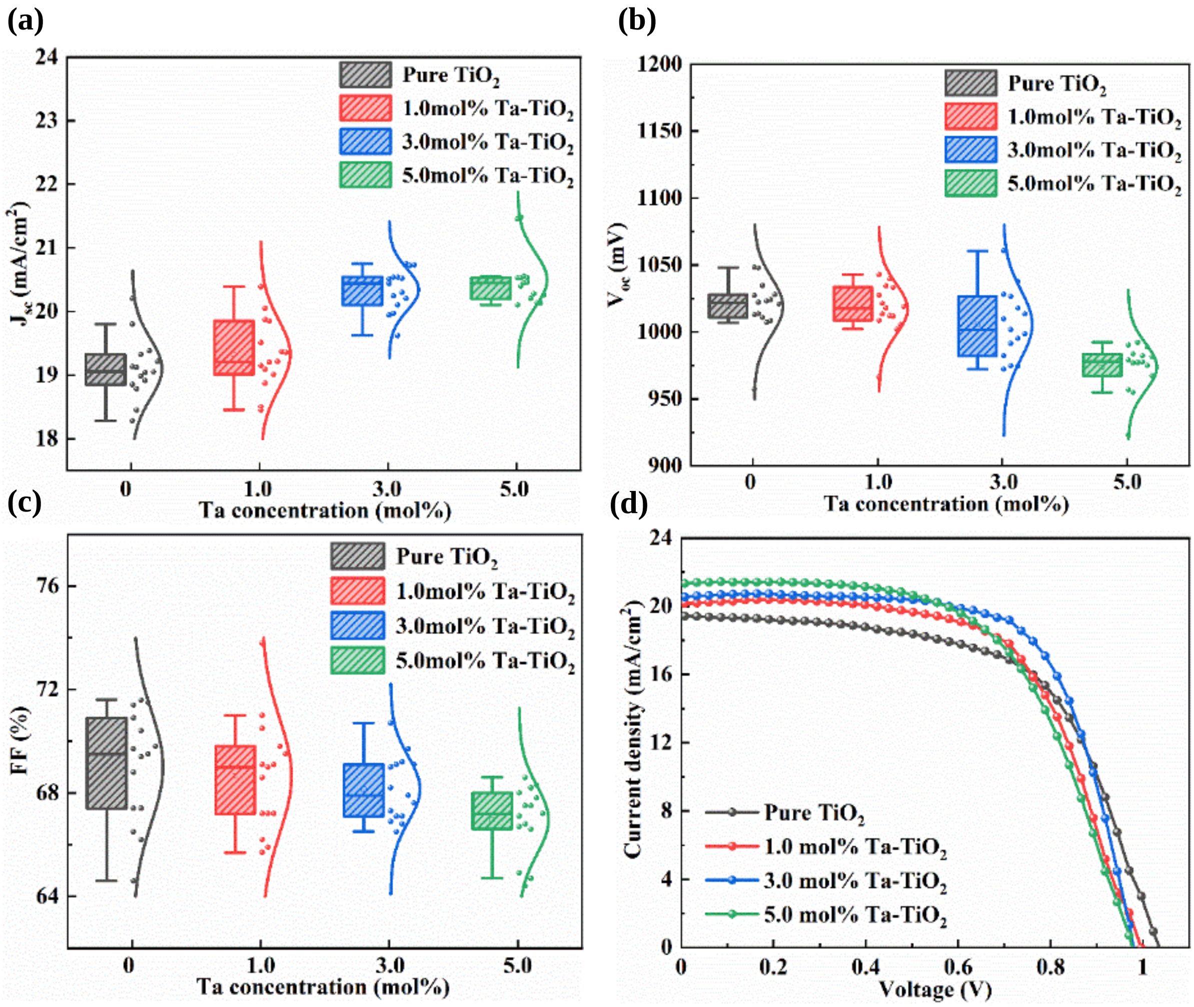

Figure S2 (a) J_{SC}, (b) V_{OC} and (c) FF distribution of air-processed planar PSCs based on pristine TiO_2 and Ta-TiO_2 films with various doping ratios. (d) Typical *J-V* curves for air-processed planar PSCs based on pristine TiO_2 and Ta-TiO_2 films with various doping ratios.

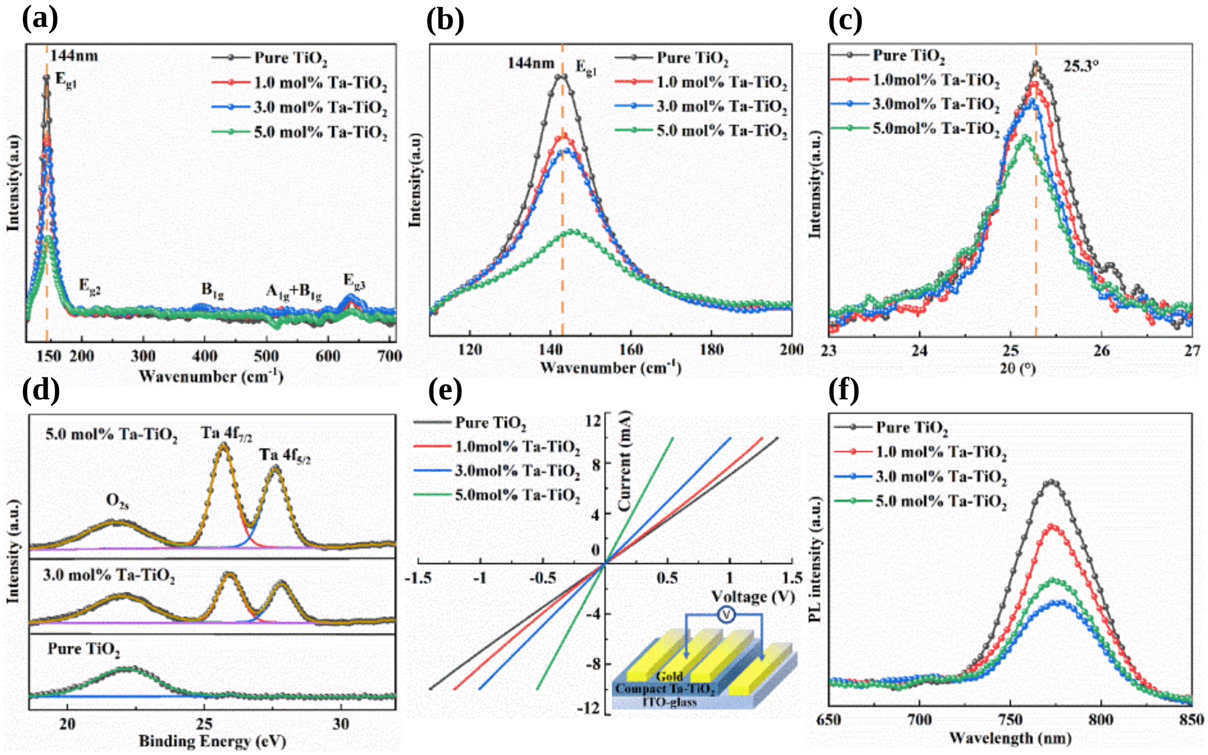

Figure S3 (a) and (b) Raman spectra of pristine TiO_2 and Ta-TiO_2 films with various doping ratios. (c) XRD patterns of pristine TiO_2 and Ta-TiO_2 films with various doping ratios. (d) High-resolution XPS spectra at Ta 4f region for pristine TiO_2 and Ta-TiO_2 films with various doping ratios. (e) *I*-*V* curve for the devices based on pristine TiO_2 and Ta-TiO_2 films with various doping ratios with an ITO/TiO_2/Au configuration. (f) Steady-state PL spectra for the perovskite films coated on the pristine TiO_2 and Ta-TiO_2 films with various doping ratios.

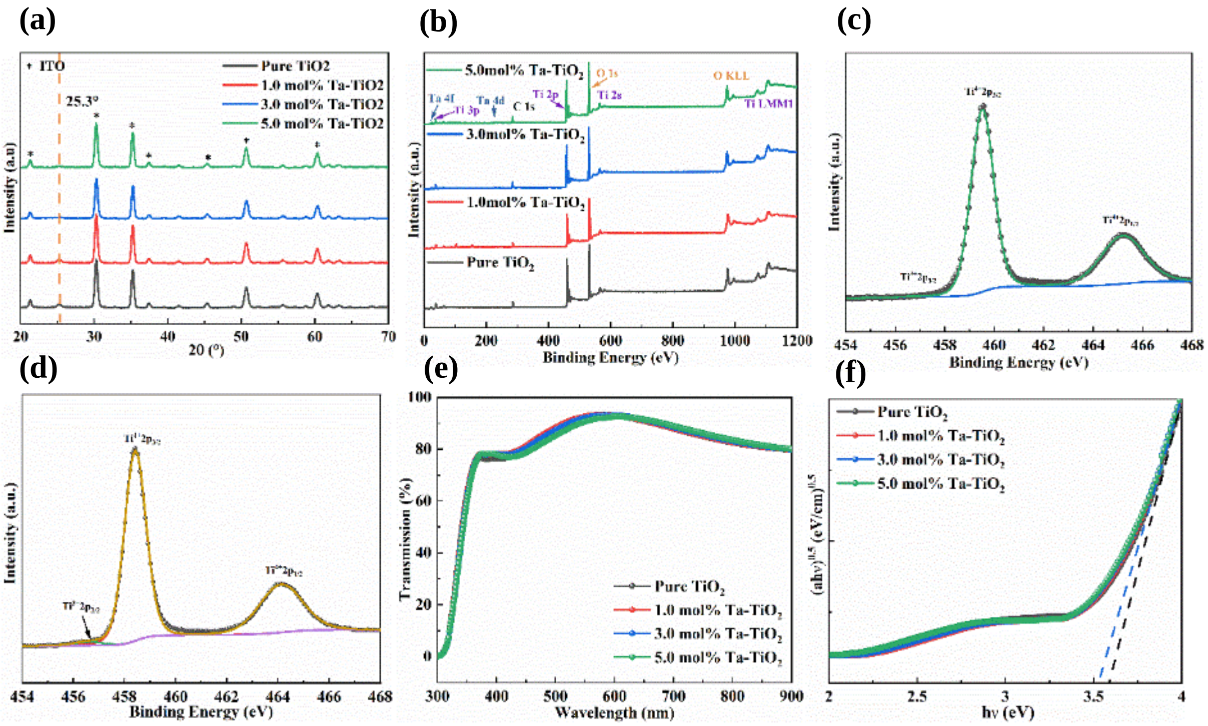

Figure S4 (a) XRD patterns of pristine TiO_2 and Ta-TiO_2 films with various doping ratios. (b) XPS survey spectra of pristine TiO_2 and Ta-TiO_2 films with various doping ratios. (c) and (d) High-resolution XPS spectra at Ti 2p region for pristine TiO_2 and 3.0mol% Ta-TiO_2 films with the $2p_{3/2}$ peaks fitted into Ti^{4+} (green) and Ti^{3+} (red) components located at 458.8 and 457.3 eV, respectively. (e) UV-Vis-NIR transmission spectra of the pristine TiO_2 and Ta-TiO_2 films with various doping ratios. (f) Tauc plots obtained from UV-Vis-NIR spectra for pristine TiO_2 and Ta-TiO_2 films.

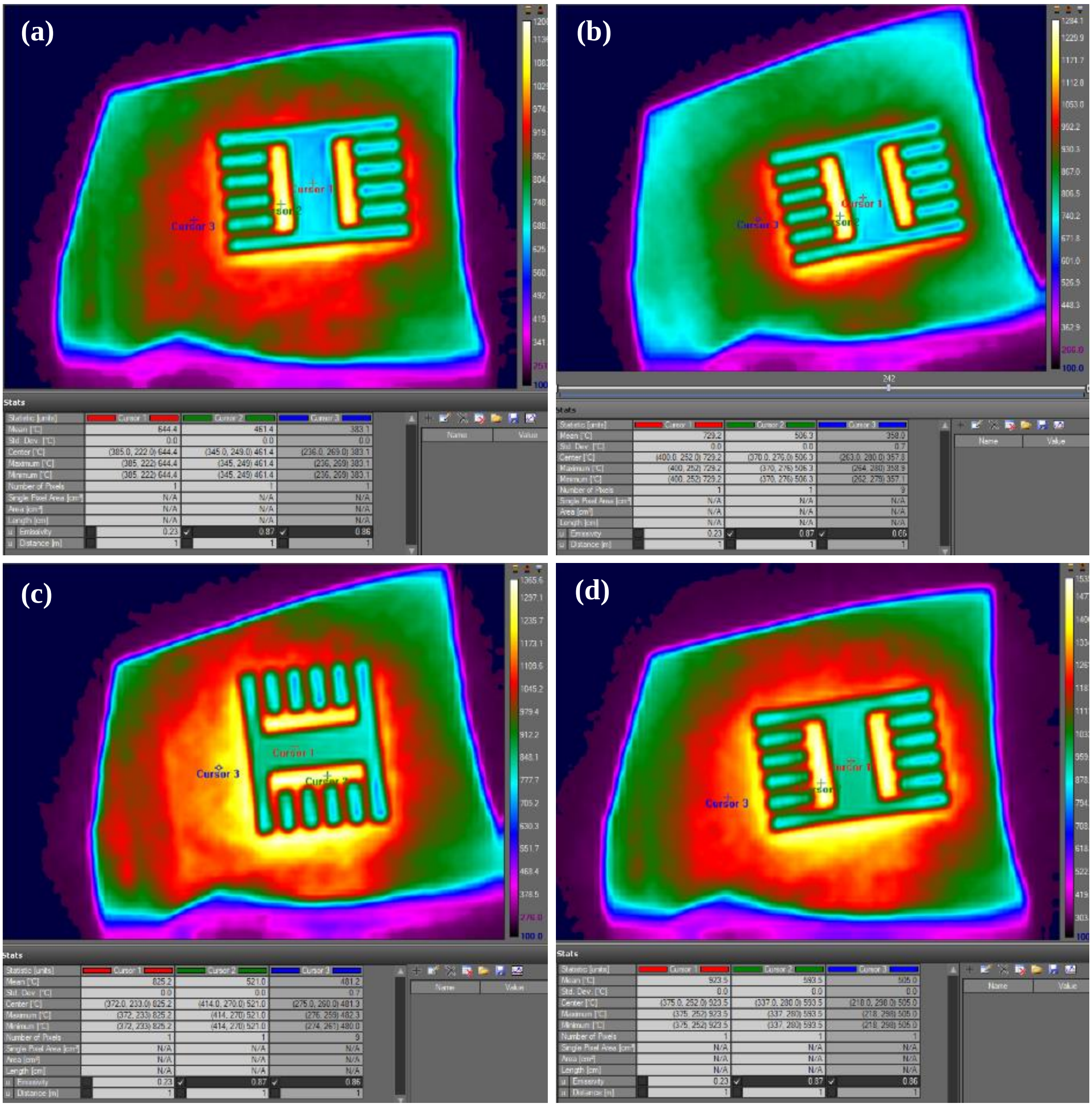

Figure S5 Thermal profiles of the Ta-TiO_2 coated on the ITO glass with the peak processing temperatures of (a) 600–650, (b) 700–750, (c) 800–850, and (d) 900–950 °C recorded by the thermal camera during the laser treatments.

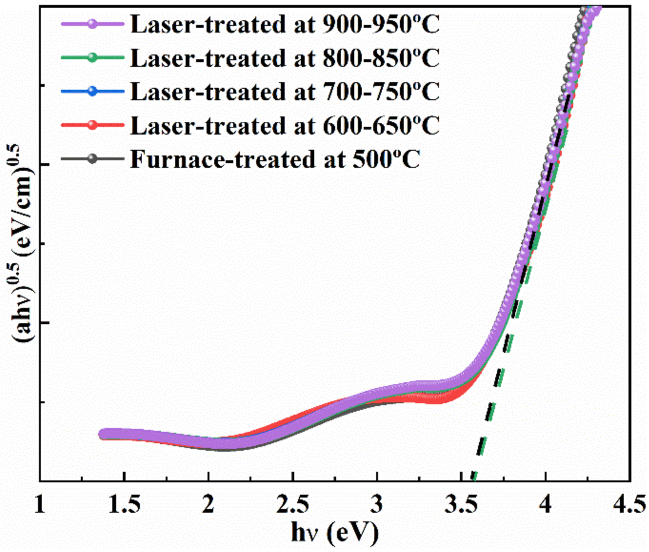

Figure S6 Tauc plots obtained from UV-Vis-NIR spectra for the furnace- and laser-treated Ta-TiO_2 films as a function of processing temperature.

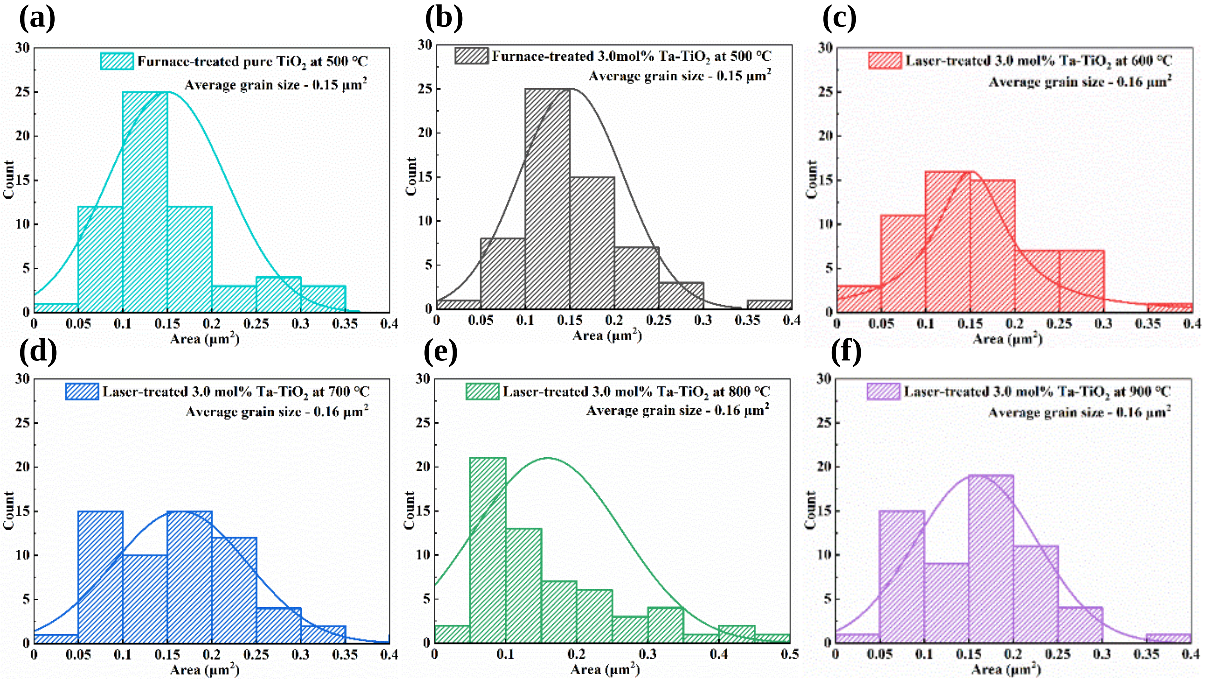

Figure S7 Grain size distribution for the perovskite films deposited on the (a) furnace-treated pristine TiO_2 film and (b) furnace-treated 3 mol% Ta-TiO_2 film, laser-treated 3 mol% Ta-TiO_2 films with the peak processing temperature of (c) 600–650 °C, (d) 700–750 °C, (e) 800–850 °C, (f) 900–950 °C.

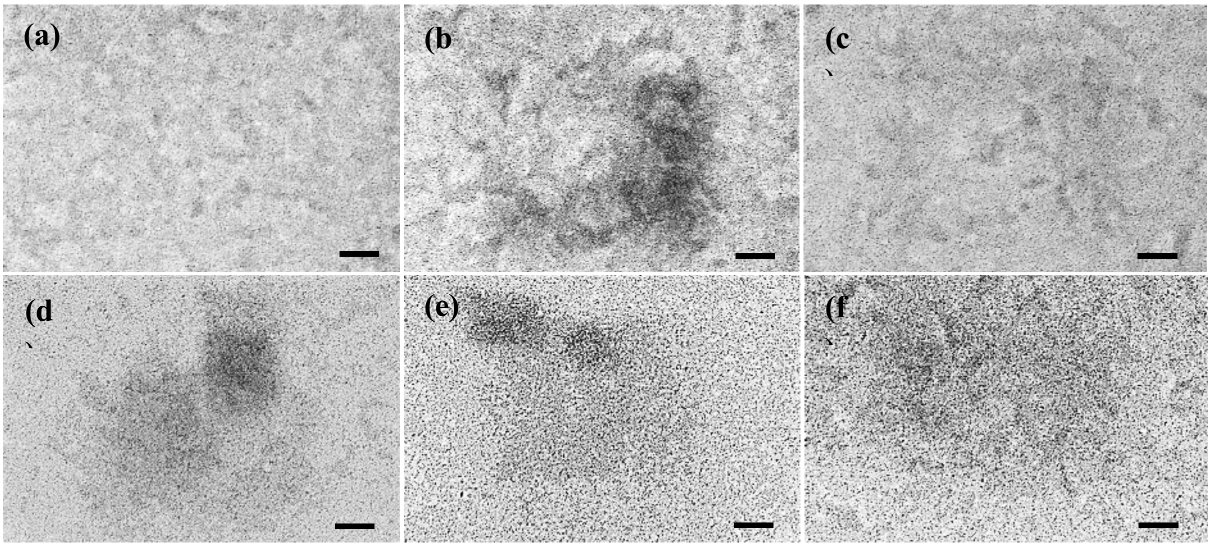

Figure S8 Top view SEM images of the (a) furnace-treated pristine TiO_2 film and (b) furnace-treated 3 mol% Ta-TiO_2 film, laser-treated Ta-TiO_2 films with the peak processing temperature of (c) 600–650 °C, (d) 700–750 °C, (e) 800–850 °C, (f) 900–950 °C (Scale bar = 200nm).

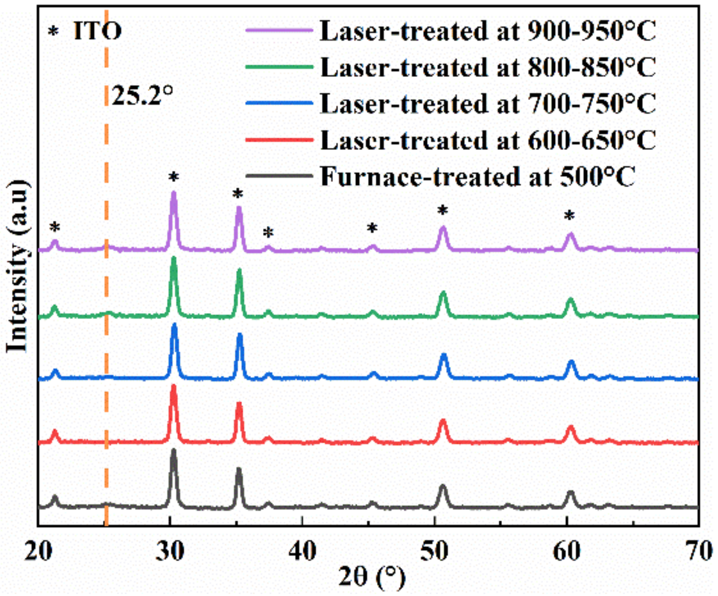

Figure S9 XRD patterns of the furnace- and laser-treated Ta-TiO_2 films as a function of processing temperature.

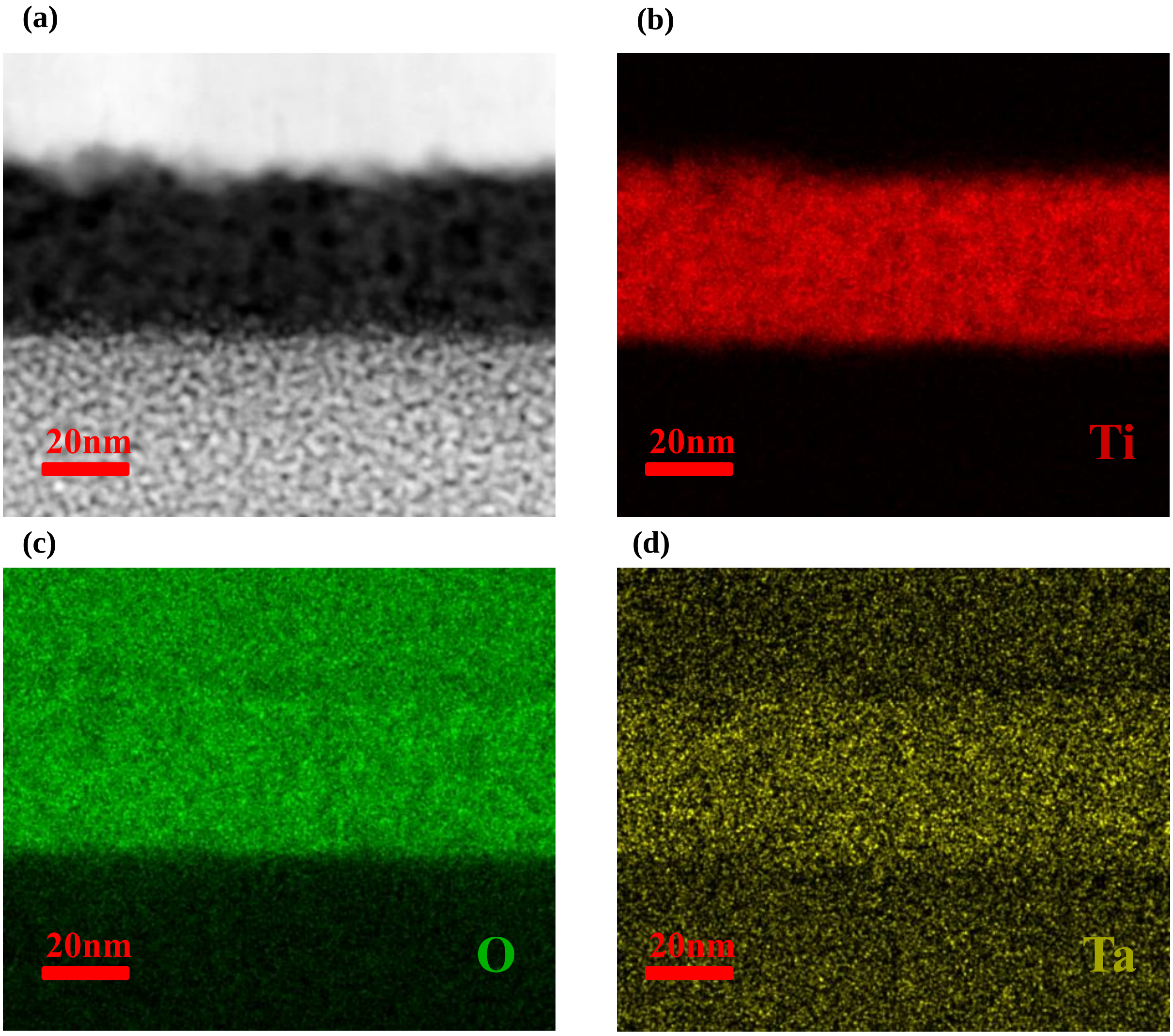

Figure S10 (a) Cross-sectional view TEM images of laser-treated Ta-TiO_2 films with the peak processing temperature of 800–850 °C. EDX elemental mapping of (b) Ti, (c) O and (d) Ta.

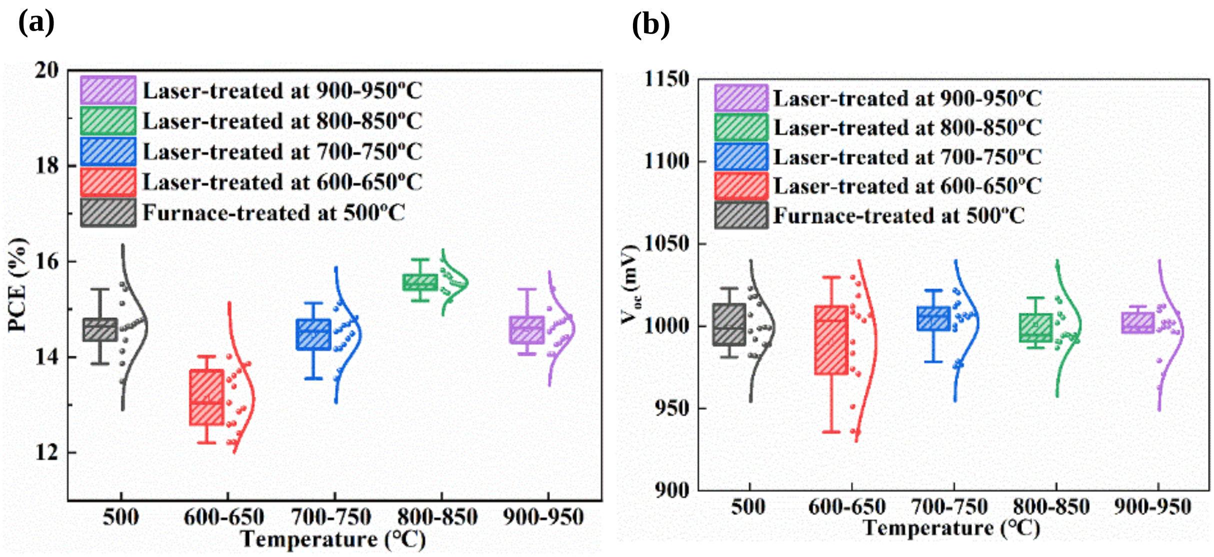

Figure S11 (a) PCE and (b) V_{OC} distribution for $CH_3NH_3PbI_3$ devices based on the furnace- and laser-treated Ta-TiO_2 films as a function of processing temperature.

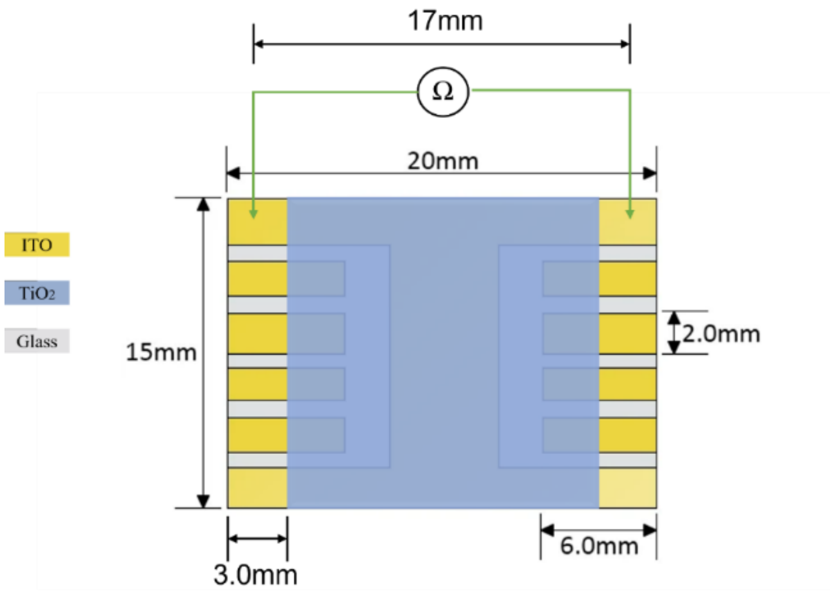

Figure S12 Schematic representation of the ITO resistance measurement with a two-probe method at a fixed distance of 17 mm between the two ITO electrodes

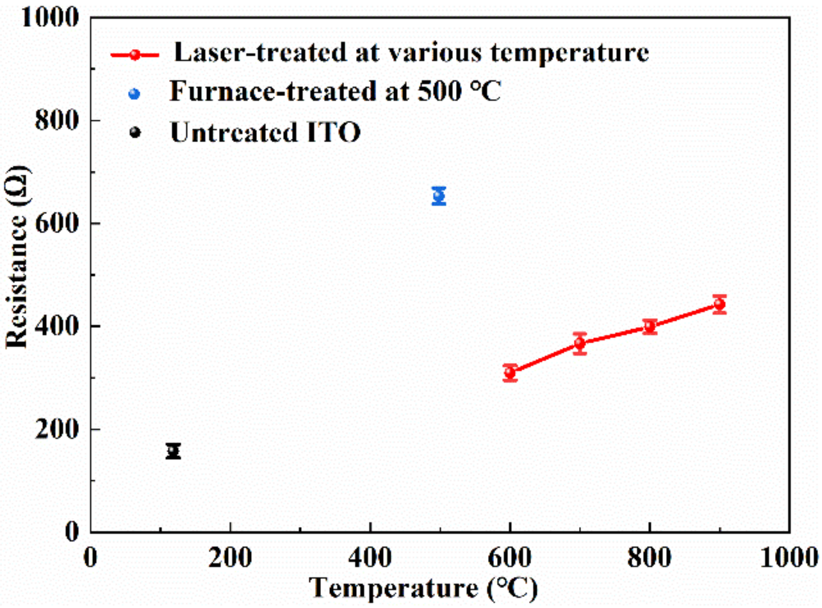

Figure S13 Resistance measurements for the untreated ITO electrodes and ITO electrodes treated using a furnace at 500 °C for 30 min and laser with various laser-induced processing temperatures.

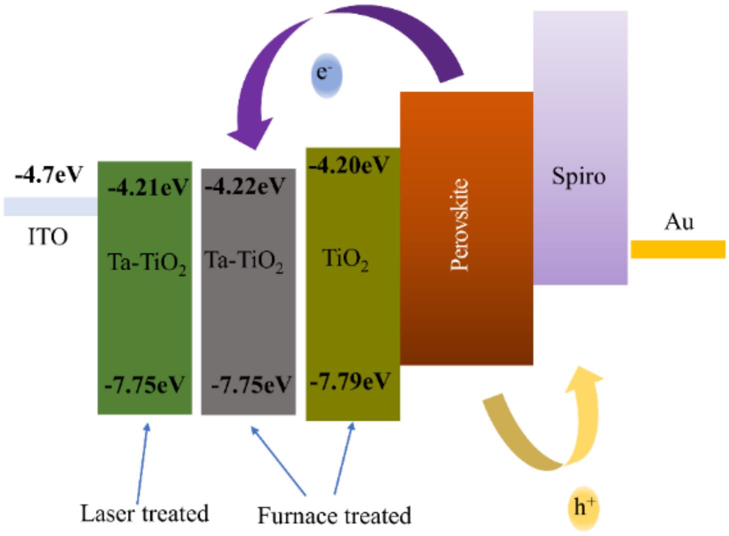

Figure S14 Band diagram of the planar PSCs based on the furnace-treated pure TiO_2 and Furnace treated and laser-treated 3.0 mol% Ta-TiO_2 ETLs

Table S1 Summary of photovoltaic parameters of the air-processed planar PSCs based on the pristine TiO_2 and Ta-TiO_2 films with various doping ratios under RH of 50-70%.

Samples	V_{OC} (V)	J_{SC} (mA·cm^{-2})	FF (%)	Average PCE (%)	Champion PCE (%)
**Pure TiO_2**	1.019±0.021	19.1±0.46	69.01±2.07	13.42±0.32	13.85
**1.0 mol% Ta-TiO_2**	1.017±0.018	19.3±0.53	68.65±2.11	13.48±0.34	14.23
**3.0 mol% Ta-TiO_2**	1.010±0.025	20.3±0.34	68.41±1.22	14.01±0.34	14.48
**5.0 mol% Ta-TiO_2**	0.975±0.017	20.5±0.41	66.97±1.32	13.40±0.29	13.79

Table S2 Summary of XRD peak position, peak intensity, FWHM, and crystalline size calculated using Scherrer equation for the pristine TiO_2 and Ta-TiO_2 films.

Samples	Peak position 2θ (°)	FWHM (°)	Peak intensity	Crystal size (nm)
**Pure TiO_2**	25.3	0.7822	723	10.41
**1.0 mol% Ta-TiO_2**	25.25	0.7925	684	10.27
**3.0 mol% Ta-TiO_2**	25.20	0.8146	620	9.99
**5.0 mol% Ta-TiO_2**	25.15	0.9552	487	8.52

Table S3 Laser parameters for the doping process of the Ta-TiO_2 films

Parameters	Spot area (cm^2)	Ramp power density (W·cm^2)	Duration (s)	Soak power density (W·cm^2)	Duration (s)
**Laser-treated at 600-650 °C**	19.6	110	13	26	3
**Laser-treated at 700-750 °C**		113	14	33	3
**Laser-treated at 800-850 °C**		113	18	36	3
**Laser-treated at 900-950 °C**		110	22	36	3

Table S4 Summary of XRD peak position, peak intensity, FWHM, and crystalline size calculated using Scherrer equation for the furnace- and laser-treated Ta-TiO_2 films as a function of the processing temperature.

Conditions	Peak position 2θ (⁰)	FWHM (⁰)	Peak intensity	Crystal size (nm)
**Furnace-treated at 500 ⁰C**	25.20	0.8146	620	9.99
**Laser-treated at 600-650 ⁰C**	25.20	0.8588	349	9.48
**Laser-treated at 700-750 ⁰C**	25.20	0.8261	541	9.85
**Laser-treated at 800-850 ⁰C**	25.20	0.8025	634	10.14
**Laser-treated at 900-950 ⁰C**	25.20	0.7792	660	10.45

Table S5 Ratios of Ti^{3+}/ Ti^{4+} and Ta^{5+}/ Ti^{4+} obtained from XPS spectra for the furnace- and laser-treated Ta-TiO_2 films as a function of the processing temperature.

Conditions	Ta^{5+}/ Ti^{4+}	Ti^{3+}/ Ti^{4+}
**Furnace-treated at 500 ⁰C**	0.056 ± 0.005	0.017 ± 0.001
**Laser-treated at 600-650 ⁰C**	0.057 ± 0.005	0.021 ± 0.001
**Laser-treated at 700-750 ⁰C**	0.058 ± 0.005	0.017 ± 0.001
**Laser-treated at 800-850 ⁰C**	0.058 ± 0.005	0.012 ± 0.001
**Laser-treated at 900-950 ⁰C**	0.063 ± 0.005	0.010 ± 0.001

Table S6 Summary of photovoltaic parameters for the air-processed $CH_3NH_3PbI_3$ devices based on the furnace- and laser-treated Ta-TiO_2 films as a function of the processing temperature.

Conditions	V_{OC} (V)	J_{SC} (mA·cm^{-2})	FF (%)	Average PCE (%)	Champion PCE (%)
**Furnace-treated at 500 ^oC**	0.999±0.013	22.33±0.41	65.55±1.75	14.62±0.51	15.52
**Laser-treated at 600-650 ^oC**	0.990±0.030	21.8±0.38	62.09±2.06	13.12±0.60	14.01
**Laser-treated at 700-750 ^oC**	1.002±0.014	21.7±0.34	66.47±1.29	14.46±0.42	15.13
**Laser-treated at 800-850 ^oC**	1.001±0.013	22.39±0.38	69.46±1.10	15.56±0.20	16.04
**Laser-treated at 900-950 ^oC**	0.997±0.014	22.42±0.48	65.34±1.79	14.59±0.35	15.42

Table S7 Summary of photovoltaic parameters for the air-processed $Cs_{0.1}FA_{0.9}PbI_3$ devices based on the furnace- and laser-treated Ta-TiO_2 films with the optimal conditions.

Conditions	V_{OC} (V)	J_{SC} (mA·cm^{-2})	FF (%)	Average PCE (%)	Champion PCE (%)
**Furnace-treated at 500 ^oC**	1.015±0.014	23.23±0.20	73.12±0.41	17.16±0.05	17.23
**Laser-treated at 800-850 ^oC**	1.031±0.002	23.26±0.01	76.04±0.27	18.24±0.09	18.34

Table S8 A summary of PCEs for recent ambient-processed PSCs in comparison to our planar PSCs

Perovskite	PCE (%)	Deposition method	Humidity (%)	Reference
**$Cs_{0.1}FA_{0.9}PbI_3$**	18.34	One-step	50-70	This work
**$MAPbI_3$**	16.04	One-step	50-70	This work
**$MAPbI_3$**	15.0	One-step	75	[1]
**$Cs_{0.15}FA_{0.85}PbI_3$**	15.56	Two-step	70	[2]
**$MAPbI_3$**	17.83	One-step	37.5	[3]
**$Cs_{0.17}FA_{0.83}PbI_{3-x}Br_x$**	16.4	One-step	70	[4]
**$MAPbI_3$**	16.1	One-step	30-60	[5]
**$MAPbI_3$**	14.66	One-step	70	[6]
**$MAPbI_3$**	17.24	One-step	40	[7]
**$FA_xMA_{1-x}PbI_3$**	10.86	Two-step	30	[8]
**$MAPbI_3$**	17	One-step	30	[9]
**$FAMAPbI_3$**	19.92	One-step	50	[9]
**$MAPbI_3$**	12.3	Slot-die	60	[10]
**$MAPbI_{3-x}Cl_x$**	13.50	Ultrasonic spray	30	[11]
**$CsFAMAPbI_3$**	10.56	Slot-die	40	[12]

Table S9 TRPL parameters of the perovskite films deposited on the different ETLs.
The average recombination lifetime (τ_{ave}) was estimated from the fitted data by biexponential decay function, the equation is as follows:

$$\tau_{avg} = \frac{\sum A_i \tau_i^2}{\sum A_i \tau_i}$$

Conditions	τ_1(ns)	A_1 (%)	τ_2(ns)	A_2 (%)	τ_{avg}(ns)
**Furnace-treated Pure TiO_2 at 500 °C**	10.19	8.68	332.56	91.32	331.62
**Furnace-treated 3.0mol% Ta-TiO_2 at 500 °C**	5.02	4.09	288.75	95.91	288.54
**Laser-treated 3.0mol% Ta-TiO_2 at 800-850 °C**	5.01	21.88	132.75	78.12	131.41

Table S10 EIS fitting parameters of the devices based on Furnace-treated pure TiO_2, 3.0mol% Ta-TiO_2 at 500ºC and Laser treated 3.0 mol% Ta-TiO_2 at 800-850 ºC in Figure 8(d).

**Conditions**	R_s **(Ω)**	R_{rec} **(Ω)**	***CPE*** **(F)**
**Furnace-treated Pure TiO_2 at 500 ºC**	5.3	651.1	1.2e-8
**Furnace-treated 3.0mol% Ta-TiO_2 at 500 ºC**	3.3	811.7	3.7e-8
**Laser-treated 3.0mol% Ta-TiO_2 at 800-850 ºC**	3.0	1578.0	3.0e-8

Table S11 Resistance of the ITO electrodes treated with furnace and laser processes as a function of the processing temperature.

**Conditions**	**Resistance(Ω)**
**Before Laser treatment**	156.63 ± 13.21
**Furnace-treated at 500 ºC**	652.27 ± 15.74
**Laser-treated at 600-650 ºC**	309.58 ± 14.42
**Laser-treated at 700-750 ºC**	366.76 ± 18.95
**Laser-treated at 800-850 ºC**	399.12 ± 12.36
**Laser-treated at 900-950 ºC**	442.54 ± 16.63

Table S12 The Calculated details for the energy level of Furnace-treated pure TiO_2, 3.0mol% Ta-TiO_2 at 500ºC and Laser treated 3.0 mol% Ta-TiO_2 at 800-850 ºC in Fig 8e and Fig 8f

**Conditions**	E_f **(eV)**	E_{onset} **(eV)**	E_g **(eV)**	Ev **(eV)**	Ec **(eV)**
**Furnace-treated Pure TiO_2 at 500 ºC**	-4.52	3.27	3.59	-7.79	-4.20
**Furnace-treated 3.0mol% Ta-TiO_2 at 500 ºC**	-4.49	3.26	3.53	-7.75	-4.22
**Laser-treated 3.0mol% Ta-TiO_2 at 800-850 ºC**	-4.55	3.20	3.54	-7.75	-4.21

Reference

1 J. Troughton, K. Hooper and T. M. Watson, *Nano Energy*, 2017, **39**, 60–68.

2 X. Xu, C. Ma, Y.-M. Xie, Y. Cheng, Y. Tian, M. Li, Y. Ma, C.-S. Lee and S.-W. Tsang, *J. Mater. Chem. A*, 2018, **6**, 7731–7740.

3 W. Zhang, Y. Li, X. Liu, D. Tang, X. Li and X. Yuan, *Chem. Eng. J.*, 2020, **379**, 122298.

4 U. D. Menda, G. Ribeiro, D. Nunes, T. Calmeiro, H. Águas, E. Fortunato, R. Martins and M. J. Mendes, *Mater. Adv.*, 2021, **2**, 6344–6355.

5 D. Di Girolamo, F. Matteocci, M. Piccinni, A. Di Carlo and D. Dini, *Sol. Energy Mater. Sol. Cells*, 2019, 110288.

6 Y. Yang, T. Chen, D. Pan, J. Gao, C. Zhu, F. Lin, C. Zhou, Q. Tai, S. Xiao, Y. Yuan, Q. Dai, Y. Han, H. Xie and X. Guo, *Nano Energy*, 2020, **67**, 104246.

7 K. Liao, J. Yang, C. Li, T. Li and F. Hao, *ACS Appl. Mater. Interfaces*, 2019, **11**, 39882–39889.

8 J. Duan, Z. Liu, Y. Zhang, K. Liu, T. He, F. Wang, J. Dai and P. Zhou, *Opt. Mater. (Amst).*, 2018, **85**, 55–60.

9 R. Xia, X.-X. Gao, Y. Zhang, N. Drigo, V. I. E. Queloz, F. F. Tirani, R. Scopelliti, Z. Huang, X. Fang, S. Kinge, Z. Fei, C. Roldán-Carmona, M. K. Nazeeruddin and P. J. Dyson, *Adv. Mater.*, 2020, **32**, 2003801.

10 Y.-C. Huang, C.-F. Li, Z.-H. Huang, P.-H. Liu and C.-S. Tsao, *Sol. Energy*, 2019, **177**, 255–261.

11 J. Su, H. Cai, X. Ye, X. Zhou, J. Yang, D. Wang, J. Ni, J. Li and J. Zhang, *ACS Appl. Mater. Interfaces*, 2019, **11**, 10689–10696.

12 C. Gong, S. Tong, K. Huang, H. Li, H. Huang, J. Zhang and J. Yang, *Sol. RRL*, 2020, **4**, 1900204.
